# Biphasic Continuous Esterification of Choline Chloride‐Based Deep Eutectic Solvents using ICHEM Technology: A Step toward New Hydrotropes with Potential Anticorrosive Properties

**DOI:** 10.1002/chem.202501702

**Published:** 2025-08-14

**Authors:** Qing Liu, Philippe Vermaut, Irene Malpartida, Julien Thiel, Christophe Len, Remi Nguyen

**Affiliations:** ^1^ ChimieParisTech, PSL Research University, CNRS Institute of Chemistry for Life and Health Sciences 11 rue Pierre et Marie Curie Paris F‐75005 France; ^2^ Sorbonne Universities, UPMC University Paris Paris F‐75006 France; ^3^ Deasyl S.A., 109 Chemin‐du‐pont‐du‐centenaire 1228 Plan‐les‐Ouates Geneva Switzerland

**Keywords:** continuous flow, corrosion inhibitors, deep eutectic solvents, esterification, mechanochemistry

## Abstract

Impact in Continuous Flow Heated Mechanochemistry (ICHEM) technology was used for the first biphasic continuous flow esterification of choline chloride‐based deep eutectic solvents (DESs) with nonmiscible acetic, hexanoic, and octanoic anhydrides, resulting in the synthesis of novel hydrotropic DESs. The reaction was first optimized in batch using acetic anhydride, and then scaled up to continuous flow in an 80 mL WAB Research Lab ICHEM reactor, achieving 90–95% yields at lab scales of 50–100 g. The physicochemical properties of the three new DESs were analyzed, revealing that esterification via the ICHEM process had minimal impact on these properties compared to the conventional batch method. Furthermore, the potential of these new hydrotropic DESs as anticorrosive agents was evaluated, demonstrating their effectiveness as corrosion inhibitors.

## Graphical abstract

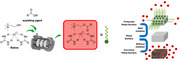

## 1 Introduction

In recent decades, deep eutectic solvents (DESs) have attracted growing interest as environmentally friendly alternatives to traditional solvents,^[^ ^1^ ^]^ owing to their advantages in safety, toxicity, cost, and processing – especially when compared to ionic liquids.^[^ ^2^ ^]^ DESs are composed of mixture of Lewis and Brønsted acids and bases, which function as acceptor and donor sites, respectively. They are characterized by a melting point lower than that of each individual component.^[^ ^1^, ^3^ ^]^ DESs are generally classified into four main types: Type I (Cat⁺X^−^ sMCl_X_), which consists of a quaternary ammonium salt and a metal chloride; Type II (Cat⁺X^−^ sMCl_X_·yH_2_O), which combines a quaternary ammonium salt with hydrated metal halides; Type III (Cat⁺X^−^ zRZ), which involves a quaternary ammonium salt with hydrogen bond donors (HBD) and Type IV (MCl_X_₋₁⁺ RZ + MCl_X_₊₁^−^), which combines hydrated metal halides and HBD.^[^ ^1a^ ^]^ Most recently, a fifth type, Type V DES, has been identified, distinguished by the fully nonionic nature of its components.^[^ ^4^ ^]^ DESs are highly effective solubilizing agents for a wide range of compounds, including carbon dioxide,^[^ ^5^ ^]^ metal oxides,^[^ ^6^ ^]^ hydrophilic organic molecules,^[^ ^7^ ^]^ and drugs.^[^ ^8^ ^]^ Consequently, they have found numerous applications in biodiesel purification, bioproduct extraction, organic synthesis, catalysis, electrochemistry, materials preparation, and metal processing.^[^ ^1^, ^3^ ^]^ To further tailoring the properties of DES, novel hydrophobic variants have been developed, using long‐chain quaternary ammonium salts or alcohols in combination with long‐chain fatty acids, which act as both HBD and hydrogen bond acceptors (HBA).^[^ ^9^ ^]^ The inclusion of aliphatic chains imparts additional properties, such as hydrotropicity, thus expanding their potential application as solvents for organic chemistry and catalysis.^[^ ^9^, ^10^ ^]^ Increasing energy consumption, dwindling fossil fuel reserves, and global environmental degradation are driving the need for new, more sustainable alternatives. Among these, efficient solvents like DESs and innovative process technologies – such as microwave chemistry,^[^ ^11^ ^]^ sonochemistry,^[^ ^12^ ^]^ mechanochemistry,^[^ ^13^ ^]^ and others – are emerging as promising solutions. These methods can be used in batch reactor or combined with continuous flow systems.^[^ ^14^ ^]^ It is obvious that the use of active, selective, and stable heterogeneous catalysts, in conjunction with continuous flow reactor and advanced technologies (e.g., continuous flow combined microwave, continuous flow combined ultrasounds, continuous flow combined ball mill (or extrusion)) should be explored at the laboratory scale. These systems have the potential to be easily adapted for industrial use.

**Scheme 1:**
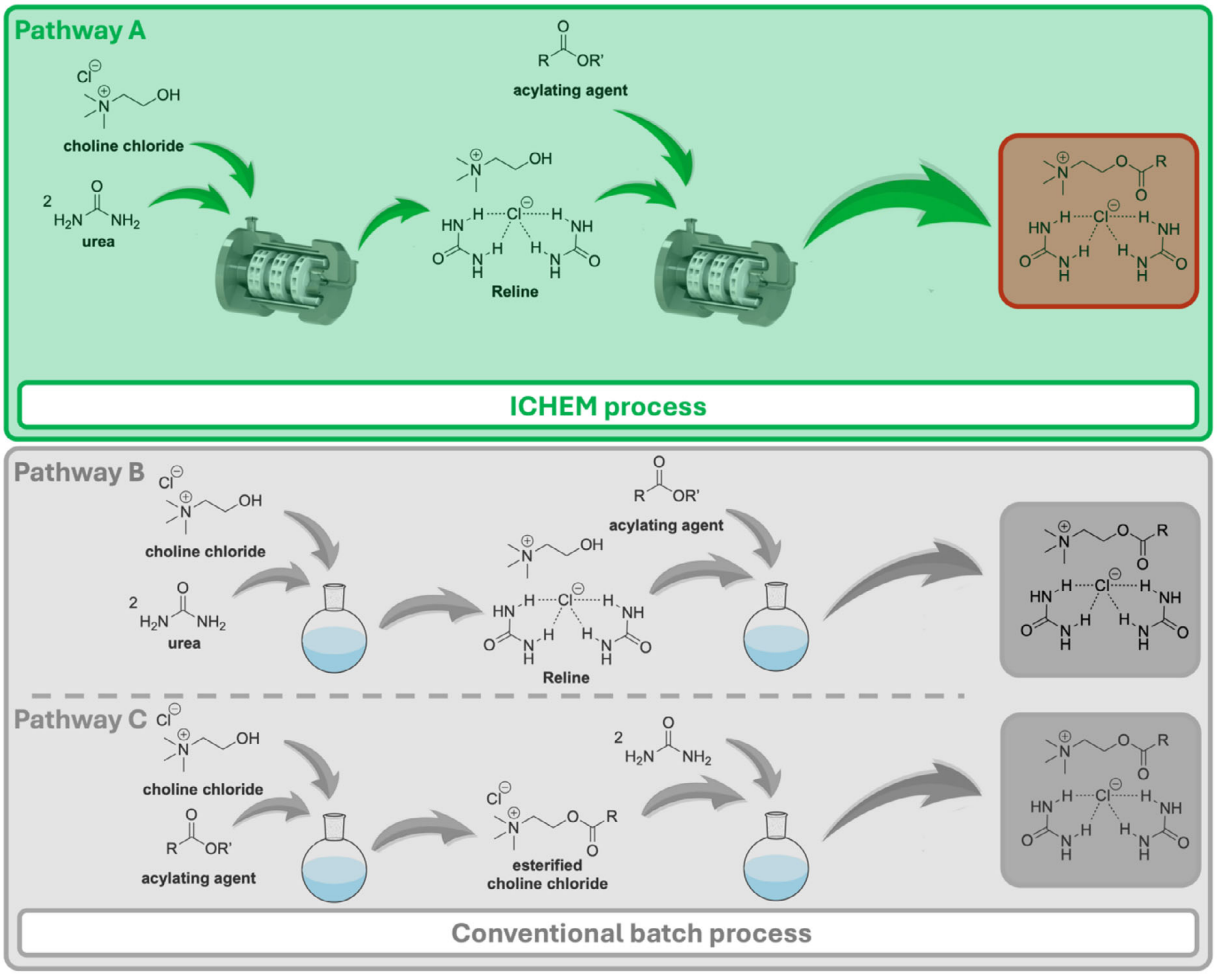
General scheme for deep eutectic solvent esterification using ICHEM technology compared with conventional pathway.

Recently, alternative mechanochemical processes have been developed for the synthesis of DESs from solid starting materials in continuous flow systems, such as extrusion^[^ ^15^ ^]^ and ICHEM (Impact Continuous Flow Heated Mechanochemistry) bead technology.^[^ ^16^ ^]^ These innovations offer several advantages, including the ability to produce DESs on demand from solid precursors, which are easier and safer to store and transport compared to their liquid counterparts. However, the hydrophilicity/lipophobicity of DESs can pose challenges in organic synthesis, particularly when reacting with lipophilic reagents, especially in continuous flow processes. Our group has addressed this challenge using ICHEM technology. For instance, we successfully produced solketal and triacetin starting from glycerol‐acetone^[^ ^17^ ^]^ and glycerol‐acetic anhydride mixtures,^[^ ^18^ ^]^ respectively, both of which are nonhomogeneous liquid‐liquid mixtures. Our group has continued to explore continuous mechanochemical synthesis of DESs and esterification, combining these processes to continuously produce both new choline acetate derivatives as HBAs before the formation of DESs, as well as new choline acetate derivatives directly as DES. In this study, we report the first aliphatic chain esterification of Reline DES (choline chloride‐urea, 1:2, v/v) with lipophilic anhydrides in a biphasic continuous flow process using ICHEM technology to produce new hydrotropic DESs. We analyzed and compared the physicochemical properties of the new hydrotropic DESs with those obtained via conventional methods (Scheme 1). Additionally, an initial evaluation of the corrosion inhibition properties of these new esterified hydrotropic DESs was conducted, suggesting their potential as effective anticorrosion agents.

## 2 Results and Discussion

### 2.1 Esterification of Choline Chloride and Reline DES in Classical Batch Reactor

Before exploring the ICHEM continuous flow pathway, initial batch studies and optimizations were conducted on the acetylation of choline chloride (ChCl) alone (Pathway C) (Table 1) and on the acetylation of choline chloride (ChCl) derived from Reline DES (ChCl‐urea, 1:2, mol/mol) (Pathway B) (Table 2), using various acylating agents. Conversions and selectivities were determined by ^1^H NMR analysis of the reaction mixtures in DMSO‐*d*_6_, while ^13^C NMR was employed to confirm the structure and composition of the esterified DES. Choline chloride hexanoate (HexChCl) and choline chloride octanoate (OctChCl) were synthesized by direct esterification of ChCl with corresponding anhydrides in a conventional batch reactor, using triethylamine as the base catalyst (Table 1).^[^ ^19^ ^]^ Acetylcholine chloride (AcChCl), on the other hand, was obtained commercially. To streamline the purification process, Et_3_N was selected as the base catalyst due to its efficient removal via vacuum evaporation or distillation, compared to other bases. For the synthesis of HexChCl, increasing the reaction time from 60 to 120 minutes in the presence of hexanoic anhydride (1.2 eq) and Et_3_N (5 mol%) resulted in a 100% yield of the target product (Table 1, entry 3). Similarly, using the same protocol for the synthesis of OctChCl, the reaction achieved 100% selectivity and 78% conversion. The lowest reactivity of octanoic anhydride prompted us to increase the amount of Et_3_N to 50 mol%, with a reaction time of 120 minutes. Under these optimized conditions, OctChCl was obtained with 100% conversion and 100% selectivity (Table 1, entry 7).

**Table 1:** Batch conventional esterification of choline chloride with hexanoic anhydride and octanoic anhydride using Et_3_N as catalyst at 65 °C (Pathway C).

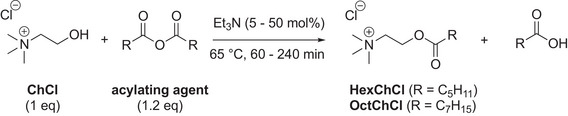					
Entry^[a]^	Acylating agent	Catalyst [mol%]	Time [min]	Conversion [%]^[b]^	Selectivity [%]^[c]^
1	(H_11_C_5_(CO))_2_O	5	60	61	100
2	(H_11_C_5_(CO))_2_O	5	90	79	74.1
3	(H_11_C_5_(CO))_2_O	5	120	100	100
4	(H_15_C_7_(CO))_2_O	5	120	78	100
5	(H_15_C_7_(CO))_2_O	10	120	82	100
6	(H_15_C_7_(CO))_2_O	10	240	89	100
7	(H_15_C_7_(CO))_2_O	50	120	100	100

^[a]^ Choline chloride (0.215 mmol), acetylating anhydride (0.257 mmol), Et_3_N (5 – 50 mol%), 65 °C, 60 – 240 minutes.

^[b]^ Conversion determined by NMR.

^[c]^ Selectivity determined by NMR.

**Table 2:** Batch conventional esterification of Reline with different anhydrides (Pathway B).

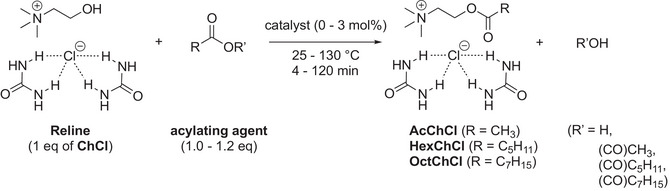						
Entry	Acylating agent	Catalyst [mol%]	Temperature [°C]	Time [min]	Conversion [%]^[c]^	Selectivity [%]^[d]^
1^[a]^	H_3_CCOOH (1 eq)	NaOH (3 mol%)	65	30	0	/
2^[a]^	H_3_CCOOH (1 eq)	NaOH (3 mol%)	65	60	0	/
3^[a]^	H_3_CCOOH (1 eq)	NaOH (3 mol%)	65	120	0	/
4^[a]^	(H_3_C(CO))_2_O (1 eq)	/	65	30	0	/
5^[a]^	(H_3_C(CO))_2_O (1 eq)	/	130	4	42	90
6^[a]^	(H_3_C(CO))_2_O (1 eq)	/	130	30	44	69
7^[a]^	(H_3_C(CO))_2_O (1 eq)	NaOH (3 mol%)	25	30	0 *^d^*	/ *^d^*
8^[a]^	(H_3_C(CO))_2_O (1 eq)	NaOH (3 mol%)	40	30	64.5	100
9^[a]^	(H_3_C(CO))_2_O (1 eq)	NaOH (3 mol%)	65	30	69.4	100
10^[a]^	(H_3_C(CO))_2_O (1 eq)	NaOH (5 mol%)	40	30	82	100
11^[a]^	(H_3_C(CO))_2_O (1.2 eq)	NaOH (5 mol%)	40	30	89.3	100
12^[a]^	(H_3_C(CO))_2_O (1.2 eq)	NaOH (5 mol%)	50	30	90.1	100
13^[a]^	(H_3_C(CO))_2_O (1.2 eq)	NaOH (5 mol%)	50	60	91	100
14^[b]^	(H_3_C(CO))_2_O (1.2 eq)	H_2_SO_4_ (5 mol%)	50	60	13	0
15^[a]^	(H_11_C_5_(CO))_2_O (1.2 eq)	NaOH (5 mol%)	50	30	67	100
16^[a]^	(H_11_C_5_(CO))_2_O (1.2 eq)	NaOH (5 mol%)	65	30	91	100
17^[a]^	(H_11_C_5_(CO))_2_O (1.2 eq)	NaOH (5 mol%)	65	60	100	100
18^[a]^	(H_15_C_7_(CO))_2_O (1.2 eq)	NaOH (5 mol%)	50	30	60.6	100
19^[a]^	(H_15_C_7_(CO))_2_O (1.2 eq)	NaOH (5 mol%)	50	60	74.6	100
20^[a]^	(H_15_C_7_(CO))_2_O (1.2 eq)	NaOH (5 mol%)	65	30	82.6	100
21^[a]^	(H_15_C_7_(CO))_2_O (1.2 eq)	NaOH (5 mol%)	65	60	96	100

^[a]^ Typical reaction conditions: Reline (“choline chloride 39 mmol”), acetylating anhydride (1 – 1.2 eq), NaOH (0 – 5 mol%), 25–130 °C, 4 – 120 minutes.

^[b]^ Typical reaction conditions: Reline (“choline chloride 39 mmol”), (H_3_C(CO))_2_O (1.2 eq), H_2_SO_4_ (5 mol%), 50 °C, 60 minutes.

^[c]^ Conversion determined by NMR.

^[d]^ Selectivity determined by NMR.

After optimizing the experimental conditions for the esterification of pure ChCl (Table 1), the esterification of Reline was explored using both basic and acid catalysts, along with different acylating agents (Table 2). Reline was prepared using a conventional protocol, where choline chloride and urea (1:2, mol/mol) were mixed in a 60 °C batch reactor until a complete liquid was observed. Under basic conditions, acetic acid did not result in significant conversion, even at 130 °C (Table 2, entries 1–3). In contrast, anhydride‐based acylation appeared to be the more suitable pathway (Table 1). When acetic anhydride was used as the acylating agent without catalyst, the reaction mixture was immiscible at 65 °C, and no conversion occured (Table 2, entry 4). However, upon increasing the temperature to 130 °C, 44% conversion was achieved after 30 minutes (Table 2, entry 6). The addition of NaOH (3 mol%) for 30 minutes resulted in a homogeneous solution and gradually increased the yield of AcChCl‐urea, with a conversion of 69% and 100% selectivity (Table 2, entries 7–9). Increasing the NaOH concentration (5 mol% versus 3 mol%), acetic anhydride (1.2 eq versus 1 eq), and temperature (50 °C versus 40 °C) led to the synthesis of AcChCl‐urea in 90% yield (Table 2, entries 10–12). Extending the reaction to 1 hour did not significantly improve the yield. It is noteworthy that these conditions, involved an excess of 20 mol% acetic anhydride and moderate heating (50 °C), as acetic anhydride can degrade to acetic acid under more severe conditions. In comparison with previous studies on polyol esterification using anhydrides,^[^ ^18^ ^]^ acidic catalysis with H_2_SO_4_ did not yield satisfactory results, and this pathway was not further investigated. This is likely due to the strong hydrogen bonding network in the Reline DES, which potentially neutralizes both Brönsted and Lewis acidity in most common DESs. The process was then extended to the use of hexanoic anhydride and octanoic anhydride to obtain the corresponding HexChCl‐urea and OctChCl‐urea, respectively. In the presence of hexanoic anhydride (1.2 eq) and NaOH (5 mol%), increasing both the temperature (65 °C versus 50 °C) and the reaction time (60 minutes versus 30 minutes) was necessary to achieve 100% yield of HexChCl‐urea (1:2, mol/mol) (Table 2, entries 15–17). Applying the same protocol with octanoic anhydride resulted in the corresponding DES with 96% yield (Table 2, entry 21). It is clear that hexanoic anhydride and octanoic anhydride were less reactive than acetic anhydride, likely due to the greater stability of these longer‐chain anhydrides, allowing for higher reaction temperature without degradation. In all cases, the initial ratio of ChCl‐urea (1:2, mol/mol) was maintained in the final DES products – AcChCl‐urea, HexChCl‐urea and OctChCl‐urea – as confirmed by NMR. To obtain pure esterified DESs, a simple purification protocol was developed. After the reaction, acetone was added, and the precipitated NaOH was filtered out. The acetone was then evaporated under reduced pressure. Next, the addition of ethylacetate resulted in the formation of two immiscible phases: one containing a solution of carboxylic acids and anhydrides in ethylacetate, and the other comprising the esterified DESs. The optimizations outlined above (Tables 1 and 2) for the esterification of choline chloride and Reline DES in a classical batch reactor were conducted as a preliminary study to gather essential informations for transferring the process to continuous flow using ICHEM technology.

### 2.2 Esterification of Reline DES in Continuous Flow Reactor Using ICHEM Technology

After optimizing the batch conditions, the reaction was transferred to a continuous flow process using ICHEM technology with WAB Research Lab equipment operating in no‐beads mode Table 3). To enable continuous flow esterification at a scale of 100 – 200 g, a large amount of Reline can be efficiently produced from choline chloride and urea through rapid continuous flow preparation of DES using ICHEM technology, as demonstrated in previous studies (see ESI).^[^ ^16^ ^]^ The esterification of Reline is carried out in a continuous flow reactor, equipped with WAB Research Lab equipment, operating without beads at a rotational speed of 8.4 m s^−1^ (Pathway A). For each acylating agents with different viscosities and reactivities, various flow rates (1.3 – 5.3 mL min^−1^) were applied to achieve optimal conversion and 100% selectivity using the WAB RL reactive cell (80 mL) (Table 3). Under the conditions used (NaOH 5 mol%, acylating agent 1.2 eq, 65 °C, residence time 15 – 60 minutes), no degradation was observed except the hydrolysis of excess anhydride, justifying the use of 120 mol% of acylating agents. Increasing the residence time from 15 minutes by decreasing the flow rate led to increased conversion: 30% for the synthesis of AcChCl‐urea (1:2, mol/mol), 13% for the synthesis of HexChCl‐urea (1:2, mol/mol), and 13% for the OctChCl‐urea (1:2, mol/mol) (Table 3). As expected, the reactivity of the anhydride decreased in the following order: acetic anhydride > hexanoic anhydride > octanoic anhydride. This trend is due to the fact that the heptyl group ((H_15_C_7_(CO))_2_O) is a stronger donor than the methyl group ((H_3_C(CO))_2_O) and the viscosity increases proportionally with the length of the aliphatic chain. Consequently, longer residence times were required for efficient conversion with hexanoic and octanoic anhydrides compared to acetic anhydride. To achieve 100% selectivity in the esterified DES at 60 – 65 °C, residence time of 30 minutes were required for the synthesis of AcChCl‐urea (1:2, mol/mol), 45 minutes for HexChCl‐urea (1:2, mol/mol), and 60 minutes for OctChCl‐urea (1:2, mol/mol). The viscosity of the resulting esterified DES increased significantly with the size of the aliphatic chain, with gel‐like solvents formed for HexChCl‐urea (1:2, mol/mol) and OctChCl‐urea (1:2, mol/mol). As observed in the synthesis of the esterified DES in conventional batch reactor, the initial ChCl‐urea ratio (1:2, mol/mol) was maintained in the final DES products – AcChCl‐urea, HexChCl‐urea and OctChCl‐urea – as confirmed by NMR. Moreover, the analysis of the NMR spectra indicates that the system is not a simple physical mixture, but rather a structured network involving hydrogen bond formation between urea and choline derivatives, accompanied by supramolecular reorganization and changes in electronic polarization (see ESI). The ^1^H NMR signal of the isolated NH group in urea shifted from 5.44 ppm to 5.58 ppm in the AcChCl—urea system and to 5.53 ppm in OctChCl—urea. Notably, no significant shift was observed in the HexChCl—urea system. This downfield shift indicates the formation of hydrogen bonds between the isolated NH group of urea and the chloride anion, leading to a decreased electron density around the hydrogen atoms. Similarly, the ^13^C NMR signal of the carbonyl group in pure urea shifted from 160.05 ppm to 160.71 ppm in AcChCl‐urea, 160.80 ppm in HexChCl‐urea, and 160.78 ppm in OctChCl‐urea. This downfield shift further supports the influence of NH···Cl^−^ hydrogen bonding on the polarization of the carbonyl double bond. Similar to NMR analysis, the IR spectra reveal specific interactions, particularly hydrogen bonding, which result in shifts of the absorption bands. For all the DESs studied, a distinct band appeared around 3190 cm^−1^, attributed to the formation of a supramolecular network involving hydrogen bonds between the amino groups of urea and the choline derivatives.

**Table 3:** Esterification of Reline with different anhydrides in continuous flow (Part of pathway A).

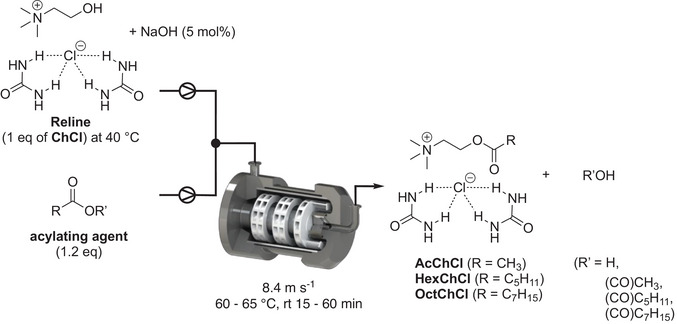					
Entry^[a]^	Acylating agent	Flow rate [mL min^−1^]	Residence time [min]	Conversion [%]^[b]^	Selectivity [%]^[c]^
1	(H_3_C(CO))_2_O	5.3	15	68	100
2	(H_3_C(CO))_2_O	2.67	30	97	100
3	(H_11_C_5_(CO))_2_O	2.67	30	83	100
4	(H_11_C_5_(CO))_2_O	1.8	45	96	100
5	(H_15_C_7_(CO))_2_O	1.8	45	74	100
6	(H_15_C_7_(CO))_2_O	1.3	60	86	100

^[a]^ Typical reaction conditions: Reline (“Choline chloride 1.0 eq”), acetylating anhydride (1.2 eq), NaOH (5 mol%), 60–65 °C, residence time 15 – 60 minutes, rotational speed 8.4 m s^−1^.

^[b]^ Conversion determined by NMR.

^[c]^ Selectivity determined by NMR.

Depending on collecting times, up to 50 – 100 g of esterified DESs could be produced using the above‐described process. These results confirm the strong performance of the ICHEM technology using WAB RL in the no‐beads operating mode, functioning as a continuous flow mixer/reactor for biphasic media. In this case, the Reline DES and aliphatic anhydride are effectively dispersed in the continuous flow, facilitating efficient esterification, thanks to the optimal geometry of the DYNO MILL accelerator.

### 2.3 Physico‐chemical Properties

After continuous synthesis using ICHEM technology (Pathway A), the physico‐chemical properties of new variety of esterified Reline DES were analyzed and compared with those of the same esterified DES obtained via the batch conventional choline chloride esterification (Pathway C) to investigate the influence of the synthetic route (Scheme 1). Several physico‐chemical properties were evaluated, including liquid state behavior, density, melting point and glass transition temperature (Table 4). Regardless of the synthetic pathway, all esterified DES exhibit strong stability in the liquid state at room temperature due to very slow nucleation. This slow nucleation is attributed to the fact that DES are mixtures, which lead to a slower nucleation process compared to pure component solvents.^[^ ^16^ ^]^ This slow nucleation phenomenon is already known and explained in the case of polymers mixture.^[^ ^20^ ^]^ This behavior is particularly emphasized in the case of our esterified DES, where the presence of aliphatic chains hinder crystallization. A gel‐like liquid is observed for HexChCl‐urea (1:2, mol/mol) and OctChCl‐urea (1:2, mol/mol) at room temperature, and even for AcChCl‐urea (1:2, mol/mol) at lower temperatures. Indeed, the aliphatic chains promote the formation of a disordered network that prevents a clean phase transition. On the other hand, these aliphatic chain‐esterified DES exhibit very high viscosities. Density was measured by weighing a known volume of DES samples in a volumetric tube, as conventional volumetric densimeters are not suitable for the highly viscous aliphatic esterified DES. For each esterified aliphatic DES, similar densities were observed for both ICHEM continuous flow and conventional batch‐processed DES. As expected, increasing the length of the aliphatic chain resulted in a lower density, due to the increased molecular spacing. Due to the low nucleation rate in DES, the melting, solidification, and crystallization points are difficult to observe using Differential Scanning Calorimetry (DSC). As a result, direct observation is required to study the melting points of DES. Because of low nucleation rate, high viscosity and dense, disordered network, the solidification and melting kinetics are very slow, and the melting point is determined through solidification points. In a sealed tube equipped with a thermometer, liquid samples were gradually cooled from 80 °C until nucleation occurred. This process enabled the determination of the melting point for each sample through two consecutive measurements. Both esterified aliphatic DESs from ICHEM continuous flow and conventional batch processes exhibited the same melting points. These melting points were also compared with those of esterified choline chloride solid compounds: AcChCl, HexChCl and OctChCl, confirming the significant reduction in melting points of DES mixtures relative to the corresponding AcChCl‐urea (1:2, mol/mol), HexChCl‐urea (1:2, mol/mol) and OctChCl‐urea (1:2, mol/mol) (mp = 95 °C – 144 °C) (Table 4). The vitreous transition refers to the shift from an organized solid state to an amorphous state, known as vitreous state, and can be analyzed through the DSC measurement curve using the graphical inflexion point method (Table 4). Additional maximum and minimum peaks may be observed in the DSC curve, but these do not offer interpretable data; therefore, only the vitreous transition temperature is discussed. Due to the dense, disordered network of aliphatic chains, all state transitions occur slowly, and the vitreous transition is delayed in both heating and cooling modes. As a result, the average vitreous transition temperature was calculated from both heating and cooling values. All vitreous transition temperatures are between ‐32 °C and ‐42 °C. Only a slight difference is observed between esterified aliphatic DESs from ICHEM continuous flow process and the conventional batch process. This difference may be due to traces of residual NaOH from ICHEM process.

**Table 4:** Physico‐chemical properties of esterified aliphatic chain DESs.

Entry	DES / component	State [25 °C]	Density [25 °C]	Melting point [°C]	Vitreous transition temperature [°C]
1	AcChCl‐urea (1:2, mol/mol)^[a]^	Full liquid	1.20	3 ± 1	−37 ± 3
2	AcChCl‐urea (1:2, mol/mol)^[b]^	Turbid liquid	1.20	2 ± 1	−32 ± 2
3	AcChCl	/	/	144 ± 2	/
4	HexChCl‐urea (1:2, mol/mol)^[a]^	Gel type liquid	1.10	31 ± 2	−37 ± 2
5	HexChCl‐urea (1:2, mol/mol)^[b]^	Gel type liquid	1.14	28 ± 1	−34 ± 4
6	HexChCl	/	/	95 ± 20	/
7	OctChCl‐urea (1:2, mol/mol)^[a]^	Gel type liquid	1.10	59 ± 1	−42 ± 2
8	OctChCl‐urea (1:2, mol/mol)^[b]^	Gel type liquid	1.10	58 ± 1	−35 ± 2
9	OctChCl	/	/	130 ± 3	/

^[a]^ ICHEM technology (Pathway A).

^[b]^ Batch conventional technology using Et_3_N (Pathway C).

**Table 5:** Solubility/miscibility tests.

			Solubility [g L^−1^]			
Entry	HBA‐HBD [1:2, mol/mol]	Water	Ethanol	Acetone	Ethyl acetate	n‐Hexane
1	ChCl‐urea	miscible	Miscible	0.5	not soluble	not soluble
2	AcChCl‐urea^[a]^	miscible	Miscible	1.8	not soluble	not soluble
3	AcChCl‐urea^[b]^	miscible	Miscible	1.5	not soluble	not soluble
4	HexChCl‐urea^[a]^	miscible	Miscible	n/a^[c]^	not soluble	not soluble
5	HexChCl‐urea^[b]^	miscible	Miscible	30	not soluble	not soluble
6	OctChCl‐urea^[a]^	miscible	Miscible	n/a^[c]^	not soluble	not soluble
7	OctChCl‐urea^[b]^	miscible	Miscible	38.5	not soluble	not soluble

^[a]^ ICHEM technology (Pathway A).

^[b]^ Batch conventional technology using Et_3_N (Pathway C).

^[c]^ Residual NaOH dust perturbs observations.

**Table 6:** Solubility of different fatty compounds in water with hydrotropes.

Entry	Substrate	Hydrotrope^[a]^	Solubility (g L^−1^)
1	lauric acid	None	0
2	lauric acid	ChCl‐urea (1:2, mol/mol)	0
3	lauric acid	AcChCl‐urea (1:2, mol/mol)	0
4	lauric acid	HexChCl‐urea (1:2, mol/mol)	0
5	lauric acid	OctChCl‐urea (1:2, mol/mol)	0
6	stearic acid	None	0
7	stearic acid	ChCl‐urea (1:2, mol/mol)	0
8	stearic acid	AcChCl‐urea (1:2, mol/mol)	0
9	stearic acid	HexChCl‐urea (1:2, mol/mol)	0
10	stearic acid	OctChCl‐urea (1:2, mol/mol)	0
11	glycerol stearate	None	15.6
12	glycerol stearate	ChCl‐urea (1:2, mol/mol)	6.5
13	glycerol stearate	AcChCl‐urea (1:2, mol/mol)	21.2
14	glycerol stearate	HexChCl‐urea (1:2, mol/mol)	38.6
15	glycerol stearate	OctChCl‐urea (1:2, mol/mol)	43.8

^[a]^ Concentration of hydrotrope: 10 g L^−1^.

### 2.4 Solubility Tests

The solubility and miscibility of synthesized esterified aliphatic DESs: AcChCl‐urea (1:2, mol/mol), HexChCl‐urea (1:2, mol/mol) and OctChCl‐urea (1:2, mol/mol) were studied in several common solvents, including water, ethanol, acetone, ethyl acetate, and n‐hexane, and compared with the nonesterified Reline DES (choline chloride‐urea, 1:2, mol/mol) (Table 5). Solubilities were measured by stirring a saturated solution of synthesized DES in the target solvent for 3 hours at 23 °C – 25 °C (controlled by thermometer), followed by 16 hours of decantation. The decantate was then filtered and evaporated to determine the solubilized weight of DES. All DESs are miscible in water and ethanol. A slight solubility was observed in acetone, which increased with the presence and length of aliphatic chain. For example, OctChCl‐urea (1:2, mol/mol) exhibited a solubility of 38.5 g L^−1^ in acetone, compared to just 0.5 g L^−1^ for ChCl‐urea (1:2, mol/mol). Aliphatic chains enhance the lipophilicity of the DES, promoting positive interactions with acetone without compromising miscibility with water. This demonstrates the balanced hydrophilic/hydrophobic nature of aliphatic OctChCl‐urea (1:2, mol/mol). None of the DES showed solubility in ethyl acetate and n‐hexane, even for the longer‐chain having octyl group. The aliphatic chains are not long enough to counterbalance the effect of electronic charges, preventing sufficient lipophilicity for these low‐polar organic solvents. Overall, no significant differences were observed between the ICHEM‐processed aliphatic esterified DES and those produced by the conventional batch process for all the physicochemical parameters studied, indicating that that ICHEM process is reliable.

### 2.5 Potential Applications

The incorporation of aliphatic chains into the structure of esterified DESs results in a balanced hydrophilicity/hydrophobicity nature, making them as potential hydrotropes. Solubility tests demonstrated that these esterified DESs exhibit hydrotropicy properties Additional solubilization tests were conducted on three selected hydrophobic fatty compounds: stearic acid, lauric acid and glycerol stearate in water, in the presence of esterified DESs: AcChCl‐urea (1:2, mol/mol), HexChCl‐urea (1:2, mol/mol) and OctChCl‐urea (1:2, mol/mol), as hydrotropes (Table 6). Table 6 shows that the solubility of lauric acid and stearic acid in water is not enhanced by the presence of ChCl‐urea (1:2, mol/mol), HexChCl‐urea (1:2, mol/mol) and OctChCl‐urea (1:2, mol/mol). However, better affinities with glycerol stearate and ChCl‐urea (1:2, mol/mol), HexChCl‐urea (1:2, mol/mol) and OctChCl‐urea (1:2, mol/mol) lead to an increase of its solubility in water, demonstrating moderate hydrotropic properties that depend on the substrate. This hydrotropic effect diminishes for DESs with shorter aliphatic carbon chains.

**Table 7:** Influence of the proportion of OctChCl‐urea (1:2, mol/mol) corrosion inhibitor.^[a]^

	Proportion of OctChCl‐urea (1:2, mol/mol)						
	0 w%	1 w%	2 w%	3 w%	5 w%	7 w%	10 w%
Mass Loss Percentage of stainless steel beads (%)	14.5 ± 2	10.0 ± 2	7.8 ± 1	7.9 ± 1	7.3 ± 1	5.0 ± 1	1.0 ± 0.5

^[a]^ 1 g of d2 stainless steel beads after 48 hours in10 mL of 0.5 M aqueous HCl.

It is well known that many hydrotropes or surfactants can act as anti‐corrosive agent by adsorbing onto metal surface and providing protection.^[^ ^21^, ^22^ ^]^ In this preliminary study, new hydrotropic esterified DESs were tested as corrosion inhibitors using a simple weight loss test of iron fillings (CAS 7439–89–6) and stainless steel 1.4112 (X90CrMoV18) 2 mm diameter beads in a corrosive medium consisting of a 0.5 M HCl solution. For the iron filling, a 17% weight loss due to corrosion was observed after 48 hours without inhibitors, accompanied by a clear orange coloration of the solution. In contrast, only a 5% weight loss was observed in the presence of 10 wt% OctChCl‐urea (1:2, mol/mol). For the d2 stainless steel beads, a 14.5% weight loss was observed without an inhibitor, accompanied by a clear green/blue coloration of the solution. In contrast, only a 1% weight loss was observed in the presence of 10wt%OctChCl‐ urea (1:2, mol/mol) (Table 7). Various inhibitor candidates were also tested under the same conditions, with weight loss data reported in Table 8. As expected, 10wt%OctChCl‐urea (1:2, mol/mol) emerged as the best candidate, showing only 1.3% weight loss.

**Table 8:** Comparison of different candidates for corrosion inhibition.[a]

Corrosion inhibitor	None	ChCl‐urea (1:2, mol/mol)	AcChCl‐urea (1:2, mol/mol)	HexChCl‐urea (1:2, mol/mol)	OctChCl‐urea (1:2, mol/mol)	Octanoic acid
Mass Loss Percentage (%)	25.9	15.1	15.4	12.6	1.3	15.3

^[a]^ 1 g of d2 stainless steel beads after 48 hours in 10 mL of 0.5 M aqueous HCl with 10 w% inhibitor.

**Figure 1:**
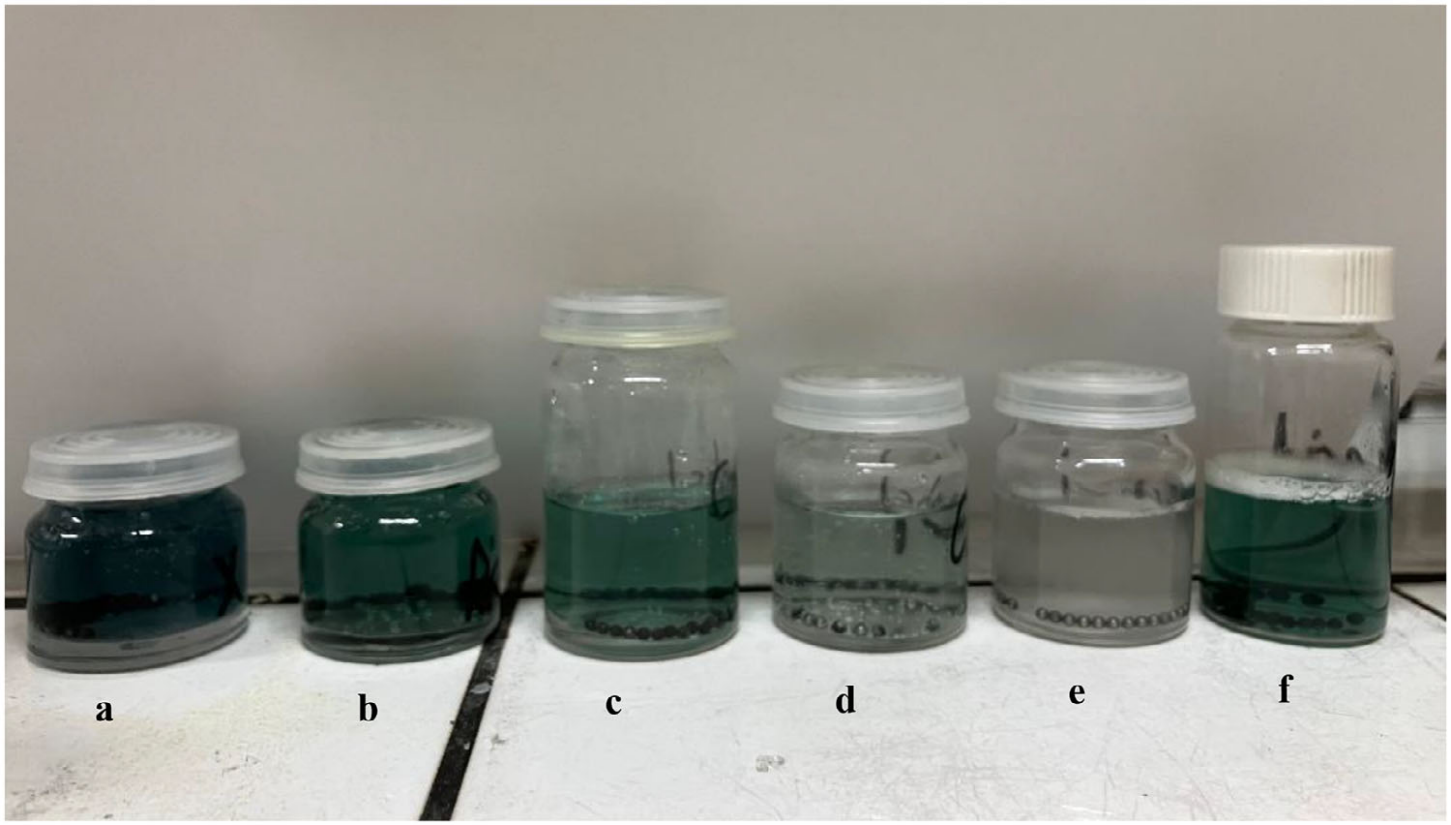
Pictures of corrosion test samples with different candidate for corrosion inhibition after 48 hours, a) no inhibitor, b) ChCl‐urea (1:2, mol/mol), c) AcChCl‐urea (1:2, mol/mol), d) HexChCl‐urea (1:2, mol/mol), e) OctChCl‐urea (1:2, mol/mol), f) Octanoic acid; sample test: 1 g of d2 stainless steel bead in 0.5 M aqueous HCl solution and 10 w% of inhibition candidate.

**Figure 2:**
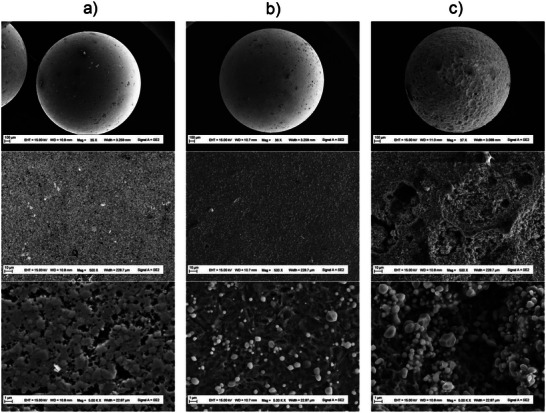
SEM images of stainless steel beads surface from corrosion tests with magnification x36, x500, x5000. a) starting stainless steel beads. b) slightly corroded stainless steel beads after exposition to 0.5 M HCl solution for 48 hours in the presence of 10 wt% OctChCl‐urea (1:2, mol/mol) inhibitor. c) highly corroded stainless steel beads after corrosion by 0.5 M HCl solution for 48 hours without inhibitor.

Reducing the length of the aliphatic chain decreases the inhibition efficiency, while unesterified ChCl‐urea (1:2, mol/mol) has no significant effect on corrosion inhibition. Octanoic acid alone has a minimal impact on corrosion inhibition, highlighting the importance of the ammonium chloride head group for efficient adsorption to the metal surface. This trend is clearly illustrated in Figure 1, which shows all sample solutions after the inhibition test, with no coloration observed for OctChCl‐urea (1:2, mol/mol) corrosion inhibitor. This observation is further supported by SEM images of the corroded bead surfaces (Figure 2).

This corrosion inhibition was confirmed through scanning electron microscopy (SEM) analysis of the d2 stainless steel beads surface after 48 hours of exposure to 0.5 M HCl corrosion media, both without inhibitors and in presence of the OctChCl‐urea (1:2, mol/mol) DES inhibitor. SEM images were compared to those of fresh d2 stainless steel beads taken at various magnifications (Figure 2a). The surface of the beads exposed to the corrosion media without any inhibitor, as seen under low and intermediate magnification (Figure 2c), shows significant galvanic pitting and crevices. Additionally, the surface is covered with small particles ranging from 1 to 5 micrometers in diameter, enriched in chromium (as confirmed by Energy Dispersive Spectroscopy EDS Figure S33). These particles are likely corrosion products formed during the dissolution of the stainless steel. In contrast, SEM images of the beads exposed to the corrosion media with OctChCl‐urea (1:2, mol/mol) DES inhibitor clearly much less steel dissolution. No pitting or crevices are visible, and while Cr‐rich particles remain, their density is substantially lower. This indicates that the inhibitor is effictive in protecting the stainless steel from corrosion in the HCl solution.

**Figure 3:**
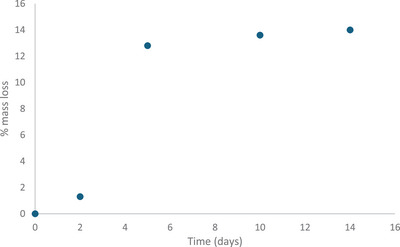
Corrosion of d2 stainless steel bead in the presence of 10 wt %OctChCl‐urea (1:2, mol/mol) inhibitor as a function of time.

In an aqueous HCl solution, a decrease in corrosion inhibition is observed after several days due to the slow hydrolytic cleavage of the ester bond. While esterified DESs remain stable in water, 30% hydrolysis of OctChCl‐urea (1:2, mol/mol) is observed after 48 hours in 0.5 M aqueous HCl solution. However, in the presence of esterified OctChCl‐urea (1:2, mol/mol) as an inhibitor, corrosion after 14 days remains lower than that observed after 2 days without the inhibitor (Figure 3). Moreover, when tap water is used as the medium, the OctChCl‐urea (1:2, mol/mol) inhibitor provides excellent corrosion protection, even after 1 month.

## 3 Conclusion

In this work, we designed and synthesized a new type of esterified aliphatic‐charged DES as hydrotropic compounds, using the first direct, sustainable esterification of ChCl‐urea (1:2, mol/mol) in continuous flow via ICHEM technology in a no‐beads operating mode. Yields of 97% was obtained for C2 esterified DES (AcChCl‐urea (1:2, mol/mol)), 96% for C6 esterified DES (HexChCl‐urea (1:2, mol/mol)) and 86% for C8 esterified DES (OctChCl‐urea (1:2, mol/mol)). *To date this esterification represents the first organic synthesis involving the transformation of a DES in continuous flow*. This method was compared with a conventional batch process, which uses more hazardous catalyst (e.g., NEt_3_). The physicochemical properties of the synthesized DESs were analyzed and compared for both processes, revealing that the ICHEM continuous flow process and the conventional batch process yield similar properties for esterified aliphatic DESs. Residual NaOH dust may require additional purification steps depending on the targeted application; however, it did not affect the properties of the esterified aliphatic DES properties in our studies. The introduction of esterified aliphatic chains imparts new and interesting physicochemical properties in terms of melting points, solubility/miscibility, electronic density, and polarity. These properties open new possibility for application in organic synthesis, materials science, and solvents. Additionally, we demonstrated the promising anti‐corrosive nature of these new hydrotropic DESs, and further in‐depth investigation of these DESs and their analogues will be conducted in future studies.

## Supporting information

The authors have cited additional references within the Supporting Information.

## Conflict of Interest

The authors declare no conflict of interest.

## Supporting information



Supporting Information

## Data Availability

The data that support the findings of this study are available in the Supporting Information of this Referencesarticle.
