# Supplementary material for: Biphasic Continuous Esterification of Choline Chloride‐Based Deep Eutectic Solvents using ICHEM Technology: A Step toward New Hydrotropes with Potential Anticorrosive Properties
Source: Chemistry. 2025 Aug 14;31(52):e01702. doi: 10.1002/chem.202501702 (PMC12444739; doi:10.1002/chem.202501702)
Supplement: Supplementary file 1 — Supporting Information [file CHEM-31-e01702-s001.docx]

**Biphasic Continuous Esterification of Choline Chloride-Based Deep Eutectic Solvents using ICHEM Technology: A Step Toward New Hydrotropes with Potential Anticorrosive Properties**

*Qing Liu,^a^ Philippe Vermaut,^b^ Irene Malpartida, ^c^ Julien Thiel,^c^ Christophe Len,^a^ Remi Nguyen,*^a^*

^a^ ChimieParisTech, PSL Research University, CNRS, Institute of Chemistry for Life and Health Sciences, 11 rue Pierre et Marie Curie, F-75005 Paris, France

^b^ Sorbonne Universities, UPMC University Paris, F-75006 Paris, France

^c^ Deasyl S.A., 109 Chemin-du-pont-du-centenaire, 1228 Plan-les-Ouates, Geneva, Switzerland.

Email: [remi.nguyen@chimieparistech.psl.eu](mailto:remi.nguyen@chimieparistech.psl.eu)

**Tables of contents**

1. General informations………………………………………………………………… S2
2. Synthesis of choline chloride derivatives..………………….………………………. S4
3. Corrosion and hydrolysis tests…………...………………….………………………. S12
4. ^1^H and ^13^C NMR spectra………………………..…………………………………… S13
5. HRMS spectra………………………..…………………………………………. S20
6. FT-IR spectra…………………………………………………………………………. S21
7. DSC curves………………………………………………………………………….. S27
8. SEM images…………………………………………………………………………. S29
9. Reference…………………………………………………………………………. S29

**General informations**

**Material.**

Choline chloride, hexanoic anhydride, octanoic anhydride and lauric acid were purchased from TCI. Acetylcholine chloride and urea were obtained from Fluorochem. Acetic anhydride and stearic acid were purchased from Acros Thermofisher. Biobased Glycerol monostearate was obtained from Moselmann. Excepted ChCl and AcChCl which were dried at 60 °C overnight before use, all products were used without further purification. All deuterated solvents were purchased from Euriso-top.

Flow reactions were carried out in a WAB DYNO^®^-MILL RESEARCH LAB equipment with a 80 mL milling cell (Fig. S1).

Fig. S1. Scheme of WAB DYNO-MILL Research lab milling cell.

**Characterizations of deep eutectic solvent.**

Proton nuclear magnetic resonance (^1^H NMR) spectra were recorded using a Bruker AC 400 (400.13 MHz). Chemical shifts are expressed in parts per million (ppm) referenced to residual DMSO (2.50 ppm). The multiplicities of the signals are reported as s (singlet), d (doublet), t (triplet), m (multiplet or overlap of non-equivalent resonances). Coupling constants (J) are given in Hz. Carbon-13 nuclear magnetic resonance (^13^C NMR) spectra were recorded using a Bruker AC 400 (100.62 MHz). The chemical shifts are expressed in parts per million (ppm) relative to the center line of the triplet at 39.52 ppm for DMSO-*d*_6_.

High-resolution mass spectroscopy (HRMS) measurements were performed using a WATERS I-Class VION IMS Q-Tof instrument equipped with an electrospray ionization (ESI) source.

FT-IR (TR) and FT-IR (ATR) spectra were recorded using the Vertex 70v FT-IR spectrometer equipped with a A225/Q Platinum ATR unit with single reflexion diamond crystal in a spectral range of 4000 cm^-1^ – 10 cm^-1^ at room temperature.

Density was measured by weighing a known volume of DES samples in a volumetric tube as usual volumetric densimeter is not convenient for those highly viscous aliphatic esterified DES.

Thermal analysis was performed using the differential scattering calorimetry (DSC) DSC3 star system equipment from Metler Toledo. Vitreous transition temperatures were determined graphically by heating sample (10-15 mg) introduced into an aluminum standard crucible of 40 µL volume. The crucible was then sealed under air and introduced into the furnace of the calorimeter along with an empty crucible of the same nature serving as a reference. DSC scans were performed in the temperature range -100 °C to 150 °C with a heating ramp of 5 °C min^-1^ and a cooling ramp of -5 °C min^-1^.

Melting points were measured as solidification points. In a sealed tube with a thermometer, liquid samples underwent a slow gradual cooling from 80 °C until nucleation. This resulted in the determination of the melting point for each sample through two consecutive measurements.

Scanning electron microscopy pictures were obtained using a Zeiss SUPRA 35 microscope operating at 15kV and equipped with a Bruker XFlash 6/60 SSD detector for Energy Dispersion Spectroscopy EDS analysis.

**Synthesis of choline chloride derivatives**

**General method for the conventional batch DES formation of Reline.** Reline was synthesized by mixing choline chloride (ChCl) (300 g, 2.15 mol, 1 eq) and urea (258 g, 4.3 mol, 2eq.) at 60 °C by hand mixing with spatula and with magnetic stirring for 4 hours until ChCl-urea (1:2, v/v) as a homogeneous liquid is obtained (m=558 g, 100%). Spectral data are in agreement with the reported literature.^1^

**^1^H NMR** (400 MHz, DMSO-*d*_6_): δ [ppm] = 3.14 (s, 9H), 3.44 (m, 2H), 3.82 (m, 2H), 5.59 (s, 8H).

**^13^C NMR** (400 MHz, DMSO-*d*_6_): δ [ppm] =160.73, 67.39, 55.58, 53.72.

**General method for the large scale fast continuous formation of Reline (10.9 g min^-1^) in a bead mill.** The continuous flow reactor was filled with 33% of zirconium oxide beads of 0.7 mm in diameter and the rotation speed was 11.1 m s^-1^. Five beakers containing dried solid ChCl (52,4 g, 0.375 mol, 1 eq) and solid urea (45 g, 0.75 mol, 2 eq) were prepared and moderately premixed with a spatula at room temperature for 30 s. Each beaker was carefully closed with parafilm paper to avoid water trapping. Then, the rotation is turned on and the solid mixture of each premixed beaker is regularly pushed to be entrained through the continuous flow reactor with the help of the driving screw to keep milling cell permanently filled during all the process. After 45 min collecting time and an output temperature of 45 °C, the target ChCl-urea (1:2, v/v) was obtained (490 g, 10.9 g min^-1^) and the structure was confirmed by ^1^H and ^13^C NMR. Spectral data are in agreement with the reported literature.^1^

**Typical experimental procedure for the synthesis of esterified Reline in classical batch reactor**

**Acetylcholine chloride-urea (1:2, mol/mol).** Reline (10 g with ChCl, 39 mmol, 1 eq) are mixed with sodium hydroxide (78 mg, 1.9 mmol, 5 mol%) in a glass flask with magnetic stirring at 50 °C. Acetic anhydride (4.8 g, 46.2 mmol, 1.2 eq) is added slowly. The mixture is stirred at 50 °C for 30 min. The mixture is cooled down to room temperature, then sodium hydroxide and solid unreacted choline chloride are removed by filtration with acetone. Unreacted anhydride and acetic acid are removed by extraction with 4 x 80 mL of ethyl acetate and then evaporation of residual ethyl acetate under vacuum afforded pure acetylcholine chloride-urea (1:2, v/v) (m=10.5 g, 90.1%).

**^1^H NMR** (400 MHz, DMSO-*d*_6_): δ [ppm] = 2.06 (s, 3H), 3.16 (s, 9H), 3.71 (m, 2H), 4.43 (m, 2H), 5.58 (s, 8 H).

**^13^C NMR** (400 MHz, DMSO-*d*_6_): δ [ppm] =170.52, 160.71, 64.07, 58.19, 53.29, 21.17.

**IR (TR):** ῦ [cm^-1^] = 3346, 3198, 3028, 2833, 1740, 1668, 1614, 1444, 1246, 1055, 958, 874, 787, 588, 536.

**IR (ATR):** ῦ [cm^-1^] = 3321, 3192, 1742, 1664, 1607, 1437, 1379, 1232, 1173, 1055, 951, 876, 789, 580, 526.

**Choline chloride hexanoate-urea (1:2, mol/mol).** Reline (10 g with ChCl, 39 mmol, 1 eq) are mixed with sodium hydroxide (78 mg, 1.9 mmol, 5 mol%) in a glass flask with magnetic stirring at 40 °C. Hexanoic anhydride (9.9 g, 46.2 mmol, 1.2eq) are added slowly. The mixture is stirred at 65 °C for 60 min. The mixture is cooled down to room temperature, then sodium hydroxide and solid unreacted choline chloride are removed by filtration with acetone. Unreacted anhydride and hexanoic acid are removed by decantation in 3 x 80 mL of ethyl acetate and then evaporation of residual ethyl acetate under vacuum afforded pure choline chloride hexanoate-urea (1:2, v/v) (m=13.9 g, 100%).

**^1^H NMR** (400 MHz, DMSO-*d*_6_): δ [ppm] = 0.87 (t, *J* = 6.8 Hz, 3H), 1.28 (m, 4H), 1.55 (m, 2H), 2.35 (m, 2H), 3.13 (s, 9H), 3.66 (m, 2H), 4.45 (m, 2H), 5.42 (s, 8H).

**^13^C NMR** (400 MHz, DMSO-*d*_6_): δ [ppm] =173.06, 160.80, 64.24, 58.07, 53.45, 33.69, 30.98, 24.22, 22.17, 14.17.

**IR (TR):** ῦ [cm^-1^] = 3383, 3196, 3022, 2958, 2872, 2496, 1740, 1668, 1614, 1485, 1317, 1248, 1171, 955, 874, 588.

**IR (ATR):** ῦ [cm^-1^] = 3333, 3188, 2957, 2872, 1736, 1668, 1616, 1421, 1246, 1167, 1107, 953, 873, 784, 582, 534.

**Choline chloride octanoate-urea (1:2, mol/mol).** Reline (10 g with ChCl, 39 mmol, 1 eq) are mixed with sodium hydroxide (78 mg, 1.9 mmol, 5 mol%) in a glass flask with magnetic stirring at 40 °C. Octanoic anhydride (12.5 g, 46.2 mmol, 1.2 eq) are added slowly. The mixture is stirred at 65 °C for 60 min. The mixture is cooled down to room temperature, then sodium hydroxide and solid unreacted choline chloride are removed by filtration with acetone. Unreacted anhydride and octanoic acid are removed by decantation in 3 x 80 mL of ethyl acetate and then evaporation of residual ethyl acetate under vacuum afforded pure choline chloride octanoate-urea (1:2, v/v) (m=13.8 g, 96%).

**^1^H NMR** (400 MHz, DMSO-*d*_6_): δ [ppm] = 0.86 (m, 3H), 1.25 (m, 8H), 1.53 (m, 2H), 2.34 (m, 2H), 3.16 (s, 9H), 3.71 (m, 2H), 4.44 (m, 2H), 5.53 (s, 8H).

**^13^C NMR** (400 MHz, DMSO-*d*_6_): δ [ppm] =173.02, 160.78, 64.24, 58.07, 53.45, 33.73, 31.50, 28.76, 24.55, 22.45, 14.32.

**IR (TR):** ῦ [cm^-1^] = 3333, 3192, 3024, 2928, 2856, 2156, 1740, 1666, 1610, 1470, 1161, 1049, 953, 876, 784, 594.

**IR (ATR):** ῦ [cm^-1^] = 3335, 3190, 2928, 2856, 1738, 1622, 1662, 1479, 1227, 1161, 1113, 1049, 953, 876, 790, 594.

**Continuous flow synthesis of esterified Reline using ICHEM technology**

**Acetylcholine chloride-urea (1:2, mol/mol).** Reline (100 g with ChCl, 0.39 mol, 1 eq) are premixed with sodium hydroxide (780 mg, 19.5 mmol, 5 mol%) at 40 °C. The mixture remains heated at 40 °C - 50 °C and pumped with a Teledyne peristaltic pump calibrated for 1.75 mL/min (*pump 1*). Pure acetic anhydride is pumped with a syringe pump calibrated for 0.92 mL/min (*pump 2*). The two flows with a total flow rate of 2.67 mL min^-1^ were passed through a T-connection and then into the WAB DYNO research Lab (internal volume = 80 mL) without bead operating mode at 8.4 m s^-1^ rotational speed. Driving screw conveyor is removed for a simple non driving screw conveyor. The cell of the 80 mL double-jacketed WAB RL reactor is heated by continuously passing water at 65 °C through a Watson Marlow peristaltic pump. An equilibration time of twice the residence time is needed to reach the steady state of the process. The reaction mixture is collected at the exit of the reactor cell for NMR analysis (97% conversion and 100% selectivity). Continuous production of acetylcholine chloride-urea is treated as described above: cooling down to room temperature and precipitating sodium hydroxide and unreacted choline chloride in acetone. After filtration, liquid-liquid extraction in ethyl acetate and then evaporation under vacuum afforded the acetylcholine chloride-urea (1:2, v/v). The structure was confirmed by ^1^H, ^13^C NMR and FT-IR.

**Choline chloride hexanoate-urea (1:2, mol/mol).** Reline DES (100 g with ChCl, 0.39 mol, 1 eq) are premixed with 780 mg of sodium hydroxide (19.5 mmol, 5 mol%) at 40 °C. The mixture remains heated at 40-50 °C and pumped with a Teledyne peristaltic pump calibrated for 0.81 mL/min (*pump 1*). Hexanoic anhydride is pumped with a syringe pump calibrated for 0.99 mL/min (*pump 2*). The two flows with a total flow rate of 1.80 mL min^-1^ were passed through a T-connection and then into the WAB DYNO research Lab (internal volume = 80 mL) without bead operating mode at 8.4 m s^-1^ rotational speed. Driving screw conveyor is removed for a non simple non driving screw conveyor. The cell of the 80 mL double-jacketed WAB RL reactor is heated by continuously passing water at 65 °C through a Watson Marlow peristaltic pump. An equilibration time of twice the residence time is needed to reach the steady state of the process. The reaction mixture is collected at the exit of the reactor cell for NMR analysis (96% conversion and 100% selectivity). Continuous production of choline chloride hexanoate-urea is treated as described above: cooling down to room temperature and precipitating sodium hydroxide and unreacted choline chloride in acetone. After filtration, solid-liquid extraction in ethyl acetate and then evaporation under vacuum afforded the choline chloride hexanoate-urea (1:2, v/v). The structure was confirmed by ^1^H, ^13^C NMR and FT-IR.

**Choline chloride octanoate-urea (1:2, mol/mol).** Reline DES (100 g with ChCl, 0.39 mol, 1 eq) are premixed with 780 mg of sodium hydroxide (19.5 mmol, 5 mol%) at 40 °C. The mixture remains heated at 40-50 °C and pumped with a Teledyne peristaltic pump calibrated for 0.5 mL/min (*pump 1*). Octanoic anhydride is pumped with a syringe pump calibrated for 0.81 mL/min (pump 2). The two flows with a total flow rate of 1.30 mL min^-1^ were passed through a T-connection and then into the WAB DYNO research Lab (internal volume = 80 mL) without bead operating mode at 8.4 m s^-1^ rotational speed. Driving screw conveyor is removed for a non simple non driving screw conveyor. The WAB RL double jacketed 80 mL reactor cell is heated with water at 65 °C with a Watson Marlow peristaltic pump. An equilibration time of twice the residence time is needed to reach the steady state of the process. The reaction mixture is collected at the exit of the reactor cell for NMR analysis (87% conversion and 100% selectivity). Continuous production of choline chloride octanoate-urea is treated as described above: cooling down to room temperature and precipitating sodium hydroxide and unreacted choline chloride in acetone. After filtration, solid-liquid extraction in ethyl acetate and then evaporation under vacuum afforded the choline chloride octanoate-urea (1:2, v/v). The structure was confirmed by ^1^H, ^13^C NMR and FT-IR.

**Typical experimental procedure for the esterification of choline chloride in classical batch reactor**

**Choline chloride hexanoate.** Choline chloride (30 g, 0.215 mol, 1 eq) are mixed with hexanoic anhydride (55.2 g, 0.257 mmol, 1.2 eq.) in a glass rounded flask with magnetic stirring at room temperature. Triethylamine (1.0 g, 10.7 mmol, 5 mol%) are added and the mixture is stirred at 65 °C for 120 min and then the mixture is cooled down to room temperature. Unreacted anhydride and hexanoic acid are removed by decantation in 3 x 200 mL of ethyl acetate and then evaporation of residual ethyl acetate and triethylamine under vacuum afforded pure choline chloride hexanoate (54.1 g, 100%).

**^1^H NMR** (400 MHz, DMSO-*d*_6_): δ [ppm] = 0.87 (m, 3H), 1.27 (m, 4H), 1.55 (m, 2H), 2.34 (m, 2H), 3.15 (s, 9H), 3.7 (m, 2H), 4.45 (m, 2H).

**^13^C NMR** (400 MHz, DMSO-*d*_6_): δ [ppm] =172.84, 64.12, 58.16, 53.31, 33.77, 31.07, 24.33, 22.24, 14.26.

**IR (TR):** ῦ [cm^-1^] =3443, 3007, 2957, 2874, 1728, 1483, 1379, 1308, 1232, 1171, 1115, 1051, 955, 920, 868, 727.

**IR (ATR):** ῦ [cm^-1^] =3387, 3014, 2957, 2864, 1738, 1485, 1383, 1248, 1169, 1109, 1042, 953, 876, 534.

**HRMS-ESI** [(M+H)^+^]: m/z calcd. for (C_11_H_24_NO_2_)^+^ : 202.1802; found, 202.1815

**Choline chloride octanoate.** Choline chloride (10 g, 71.6 mmol, 1 eq) are mixed with octanoic anhydride (23.2 g, 85.9 mmol, 1.2eq.) in a glass rounded flask with magnetic stirring at room temperature. Triethylamine (36.2 mg, 36 mmol, 0.5 eq.) are added and the mixture is stirred at 65 °C for 120 min and then the mixture is cooled down to room temperature. Unreacted anhydride and hexanoic acid are removed by decantation in 3 x 200 mL. of ethyl acetate and then evaporation of residual ethyl acetate and triethylamine under vacuum afforded pure choline octanoate (18.9 g, 100%).

**^1^H NMR** (400 MHz, DMSO-*d*_6_): δ [ppm] = 0.86 (m, 3H), 1.26 (m, 8H), 1.54 (m, 2H), 2.34 (m, 2H), 3.15 (s, 9H), 3.69 (m, 2H), 4.45 (m, 2H).

**^13^C NMR** (400 MHz, DMSO-*d*_6_): δ [ppm] =172.84, 64.12, 58.16, 53.31, 33.81, 31.57, 28.85, 24.66, 22.50, 14.41.

**IR (TR):** ῦ [cm^-1^] =3446, 3393, 3252, 3015, 2928, 2854, 2134, 1743, 1643, 1483, 1420, 1389, 1321, 1275, 1227, 1176, 1120, 1036, 956, 877, 769, 723, 507.

**IR (ATR):** ῦ [cm^-1^] =3375, 2926, 2856, 1736, 1483, 1381, 1227, 1165, 1113, 953, 876, 722, 529.

**HRMS-ESI** [(M+H)^+^]: m/z calcd. for (C_13_H_28_NO_2_)^+^ : 230.2115; found, 230.2130

**Typical experimental procedure for the formation of esterified DES in classical batch reactor**

**Acetyl choline chloride-urea (1:2, mol/mol).** Acetyl choline chloride-urea (1:2, v/v) was synthesized by mixing acetylcholine chloride (45.4 g, 0.25mol, 1 eq) and urea (30 g, 0.5 mol, 2eq) at 40 °C by hand mixing with spatula and with magnetic stirring until acetyl choline chloride-urea (1:2, v/v) as a homogeneous liquid was obtained.

**Choline chloride hexanoate-urea (1:2, mol/mol).** Choline chloride hexanoate-urea (1:2, v/v) was synthesized by mixing of choline chloride hexanoate (29,7g, 0.125mol, 1 eq) and urea (15 g, 0.25 mol, 2 eq.) at 40 °C by hand mixing with spatula and with magnetic stirring until choline chloride hexanoate-urea (1:2, v/v) as a homogeneous liquid was obtained.

**Choline chloride octanoate-urea (1:2, mol/mol).** Choline chloride octanoate-urea (1:2, v/v) was synthesized by mixing choline chloride octanoate (24 g, 0.09 mol, 1 eq) and urea (10.8 g, 0.18 mol, 2 eq.) at 40 °C by hand mixing with spatula and with magnetic stirring until choline chloride octanoate-urea (1:2, v/v) as a homogeneous liquid was obtained.

**Corrosion and hydrolysis tests**

**Corrosion tests.** A mixture of metal sample (1 g, stainless steel 1.4112 (X90CrMoV18) 2 mm diameter beads) and aqueous solution of HCl (0.5 M, 10 mL) with appropriate amount of inhibitor was studied. After 48 hours, the solution is filtered, and the corroded metal is washed successively with distilled water and acetone. The corrosion state is determined by weighting the metal after 2 h in oven at 60°C. This corrosion test was repeated starting from iron fillings (1 g, CAS 7439-89-6).

**Choline chloride octanoate-urea (1:2, mol/mol) hydrolysis test.** Choline chloride octanoate-urea (1:2, v/v) (100 mg, 0.26 mmol) are added to aqueous solution of HCl (0.5 M, 10 mL) and 10 mL of water in a second test, and let resting for 48 hours at room temperature. Choline chloride octanoate-urea (1:2, v/v) remained intact in pure water test. In 0.5 M hydrochloric acid solution, extraction with ethyl acetate revealed 11 mg of octanoic acid (0.08 mmol) corresponding to 30% hydrolysis.

**^1^H and ^13^C NMR spectra**


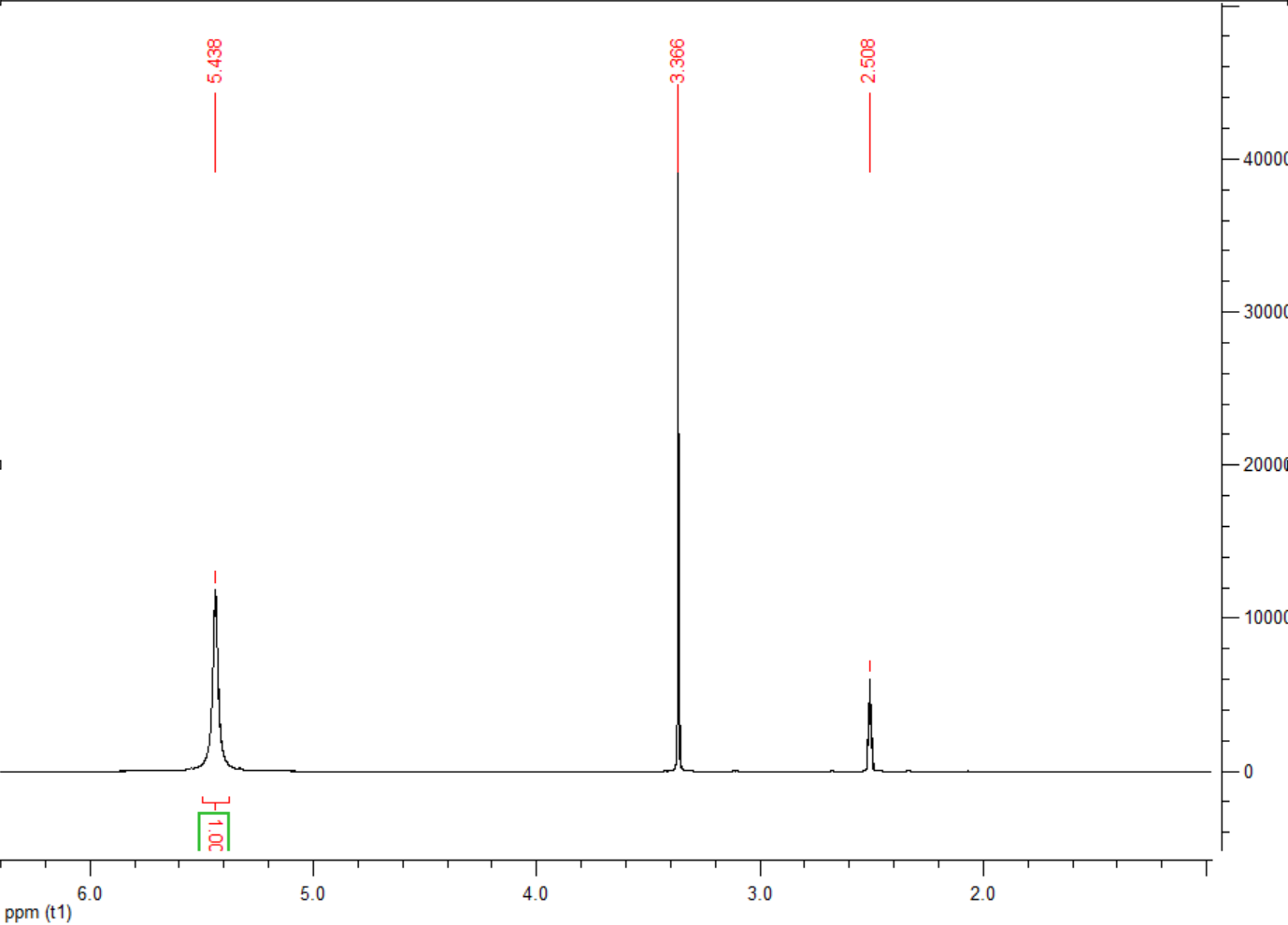


Fig. S2. ^1^H NMR of urea.


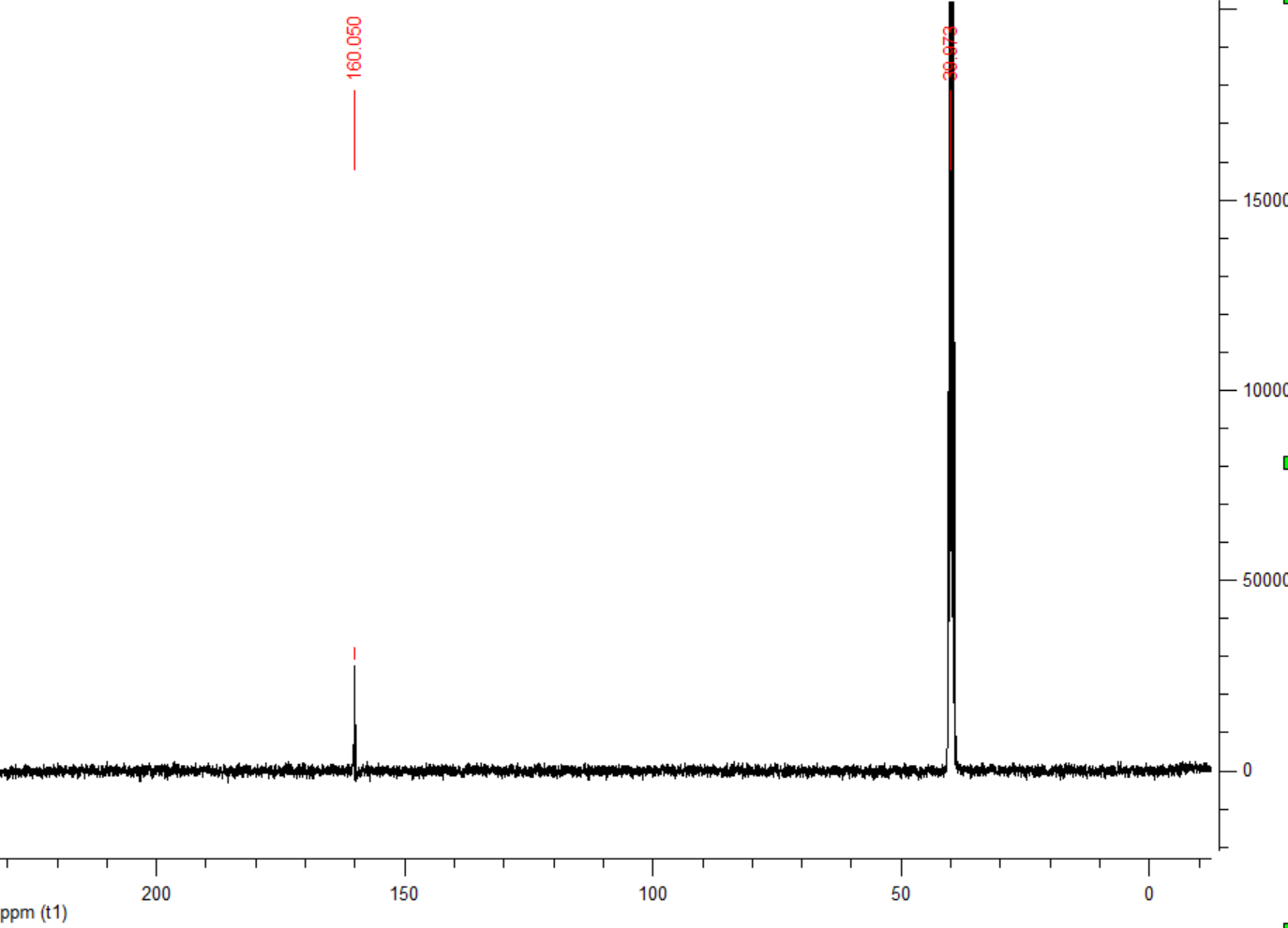


Fig. S3. ^13^C NMR of choline urea


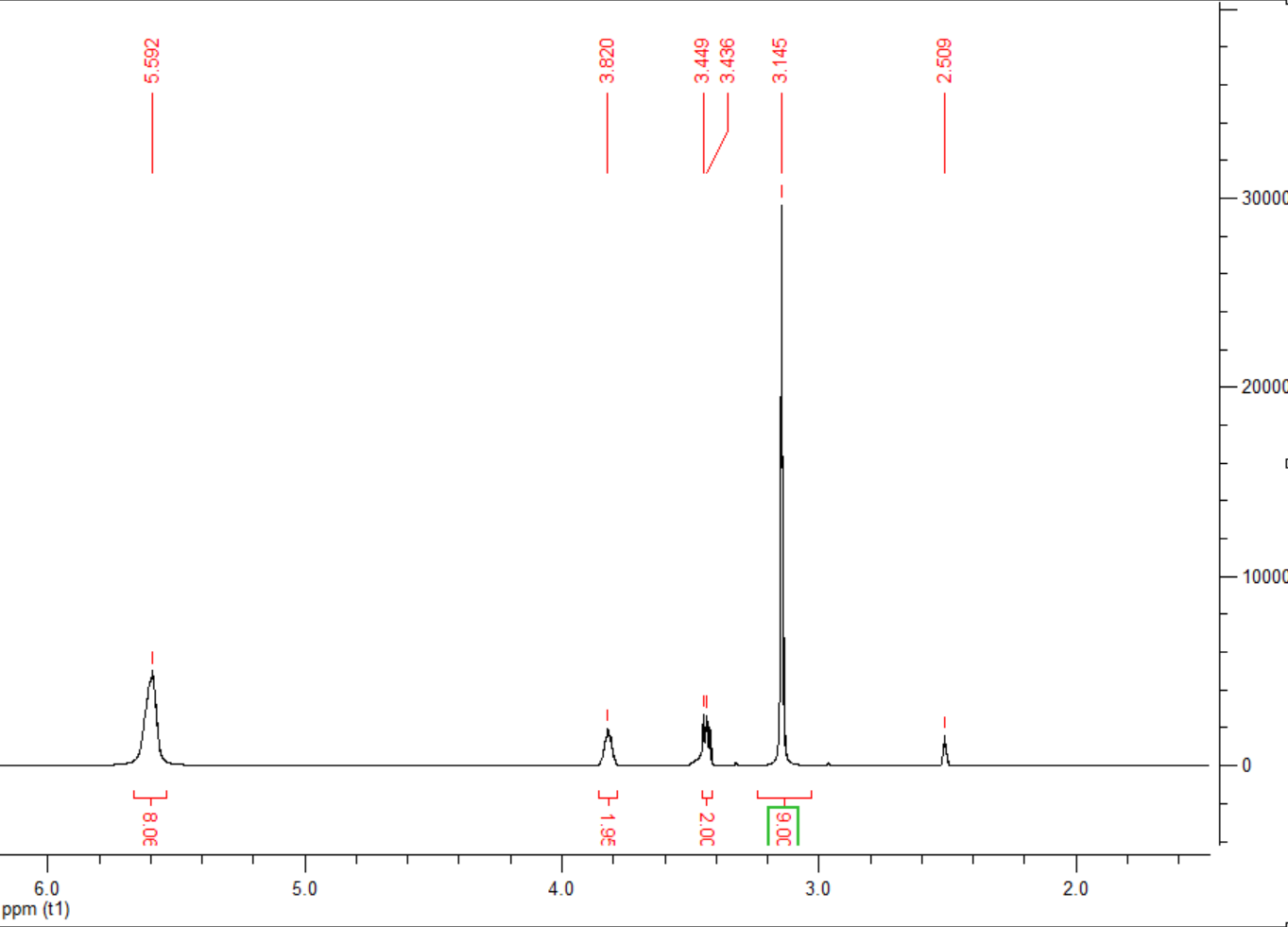


Fig. S4. ^1^H NMR of choline chloride-urea (1:2, mol/mol).


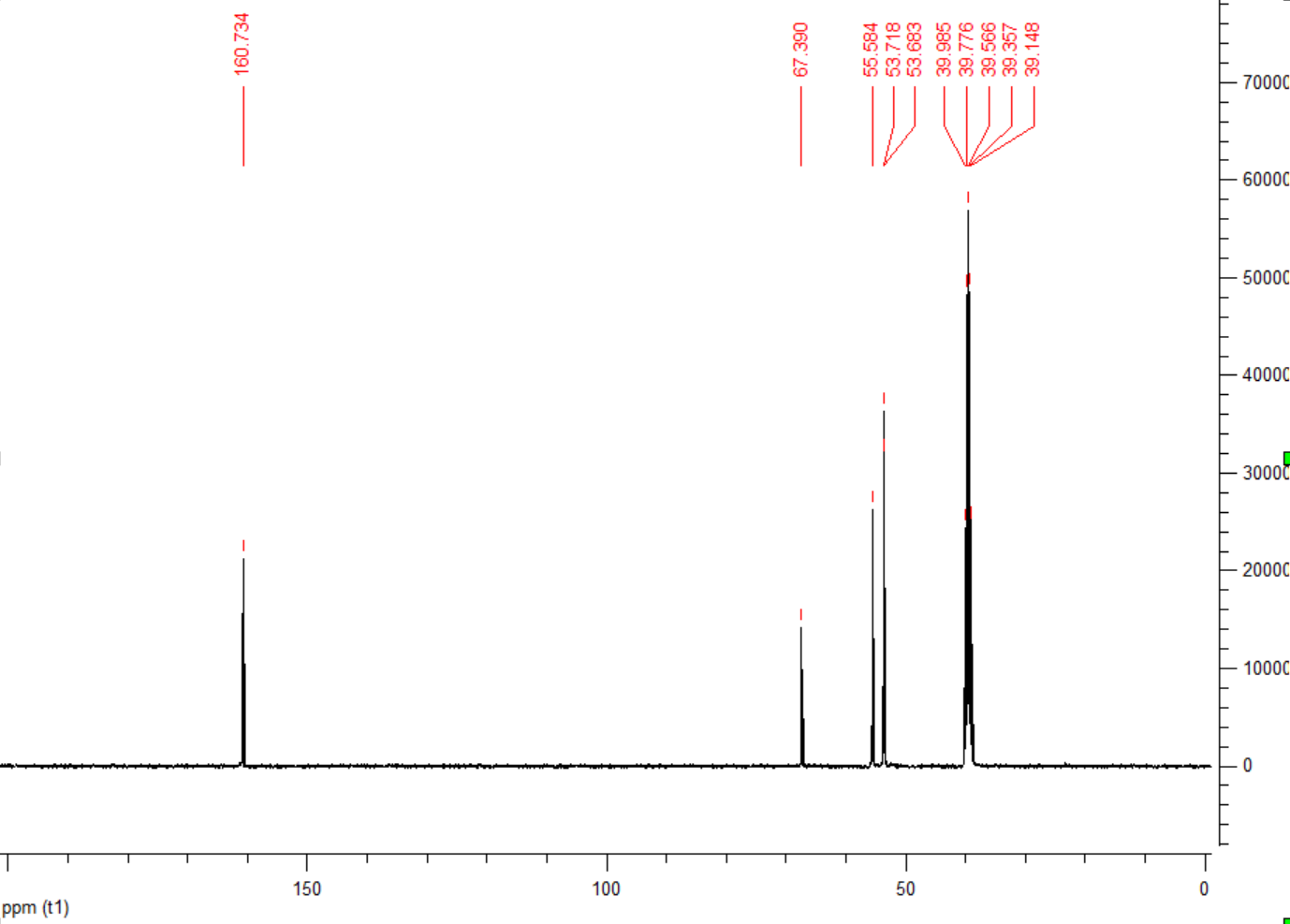


Fig. S5. ^13^C NMR of choline chloride-urea (1:2, mol/mol).


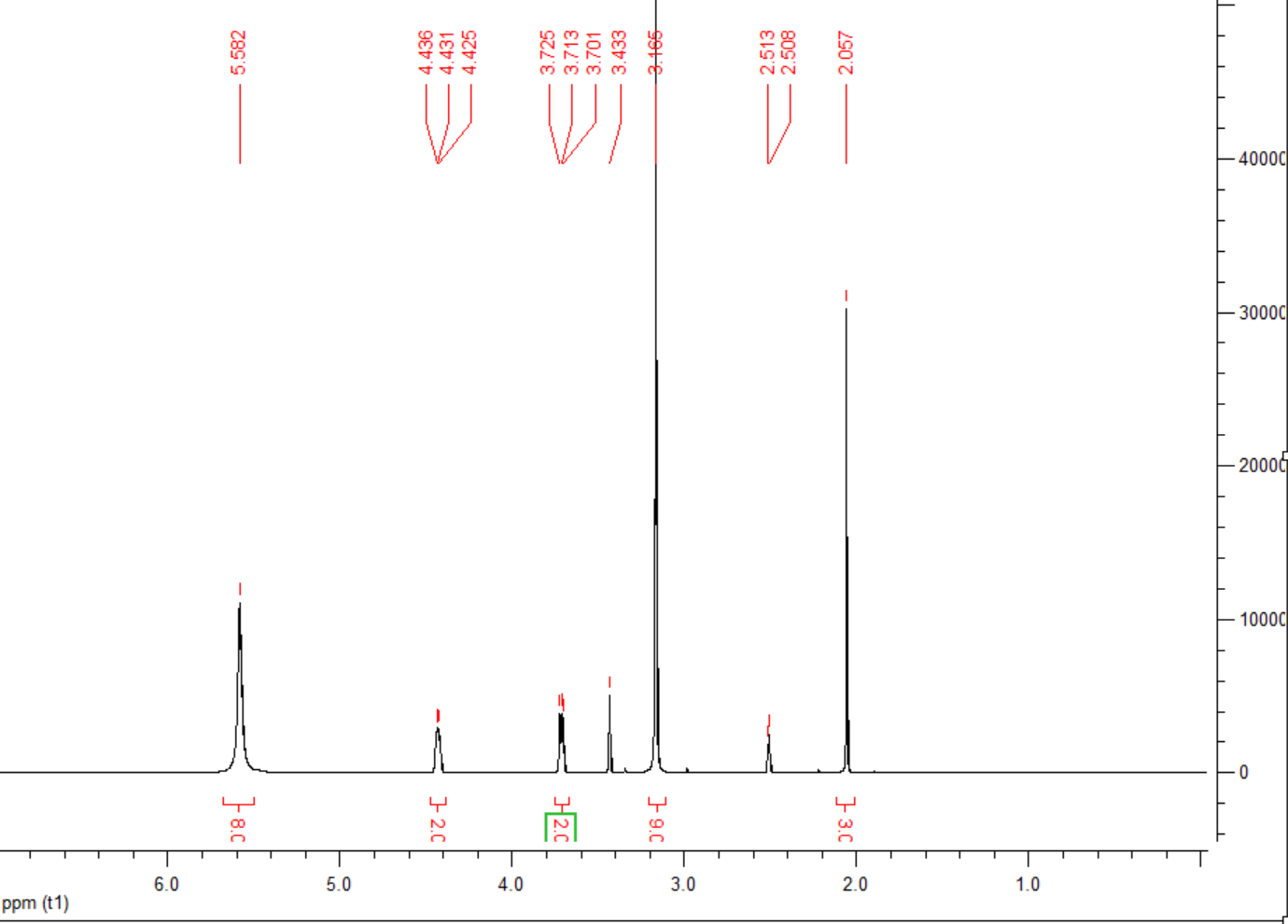


Fig. S6. ^1^H NMR of acetylcholine chloride-urea (1:2, mol/mol).


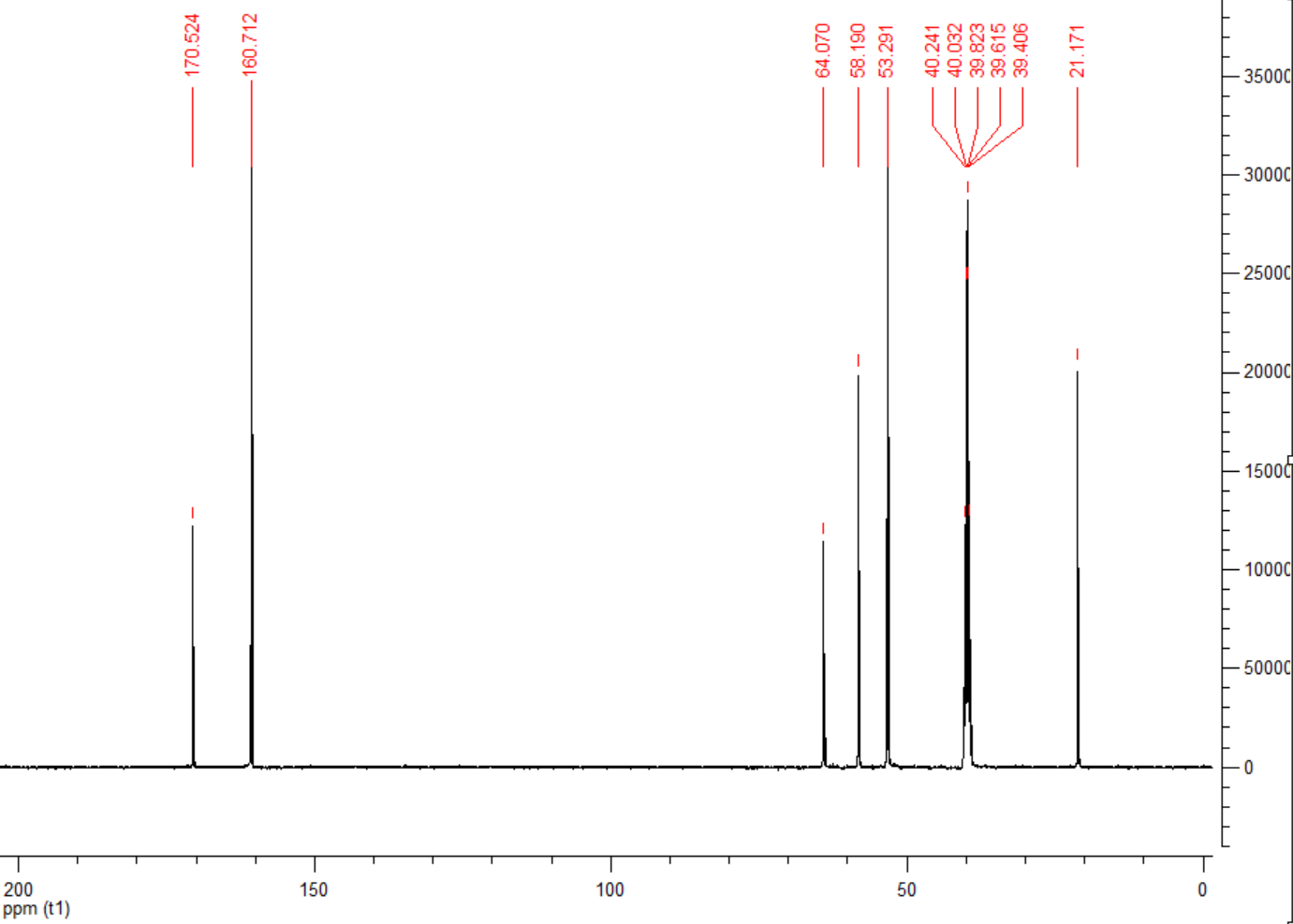


Fig. S7. ^13^C NMR of acetylcholine chloride-urea (1:2, mol/mol).


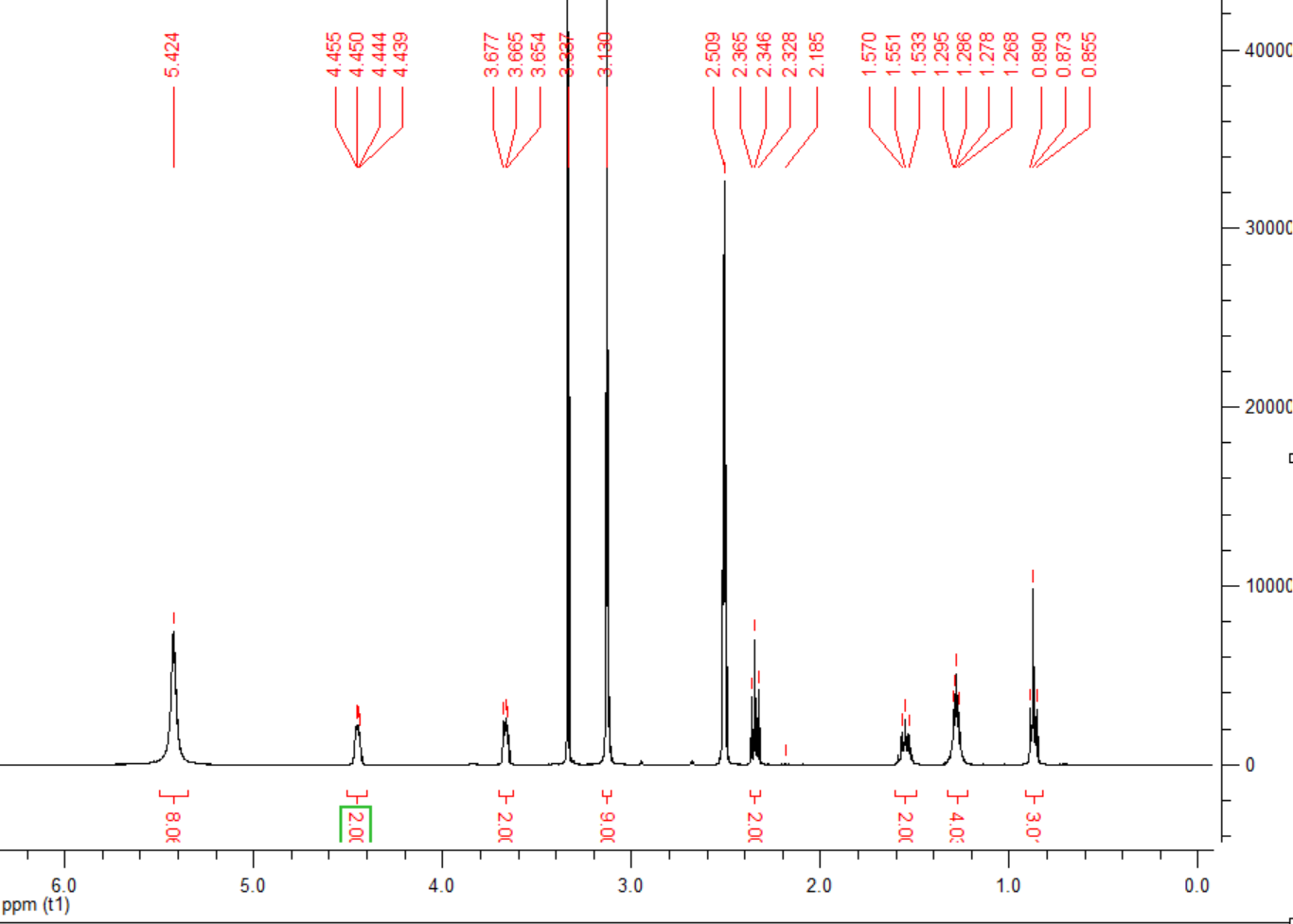


Fig. S8. ^1^H NMR of choline chloride hexanoate-urea (1:2, v/v).


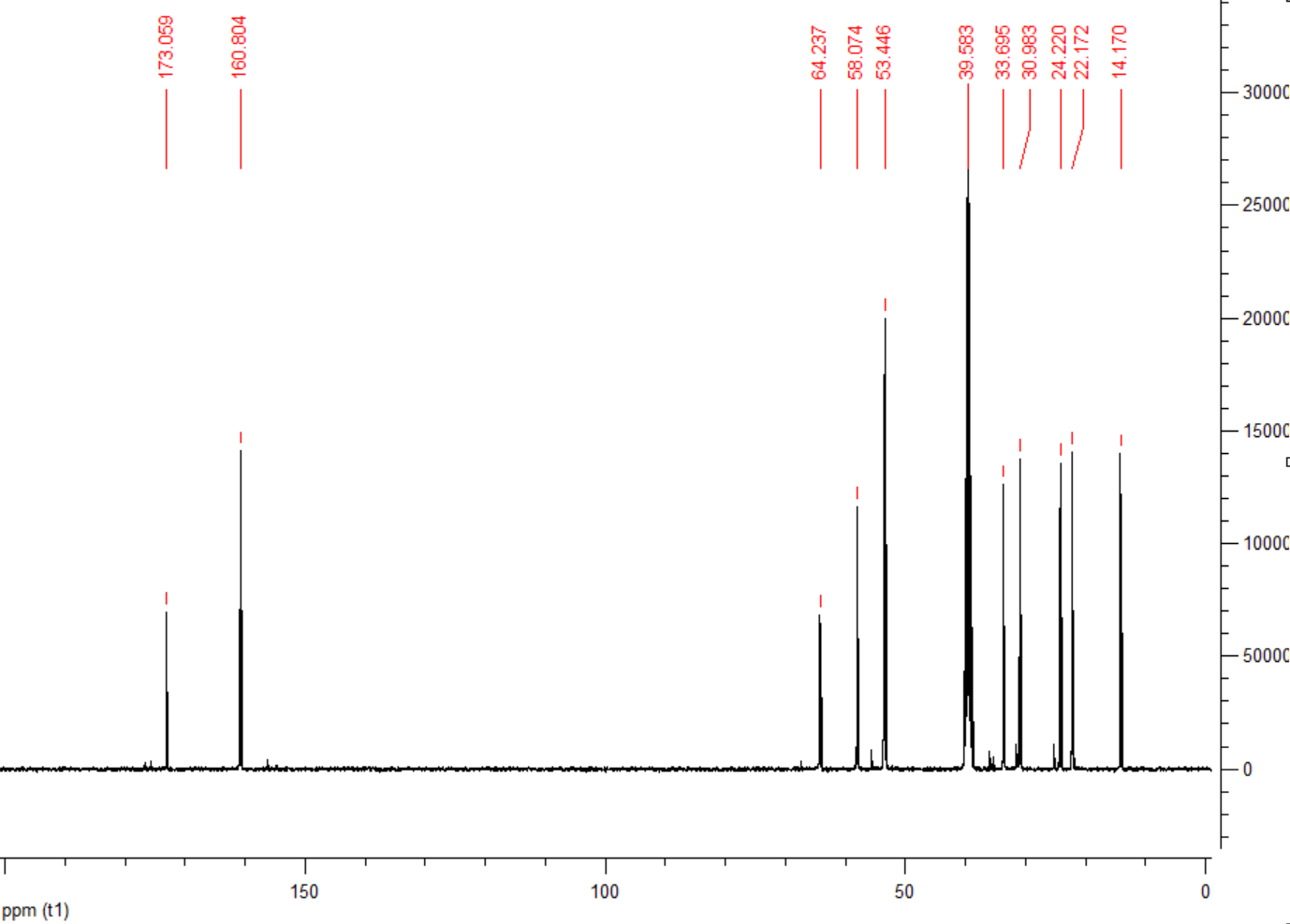


Fig. S9. ^13^C NMR of choline chloride hexanoate-urea (1:2, v/v).


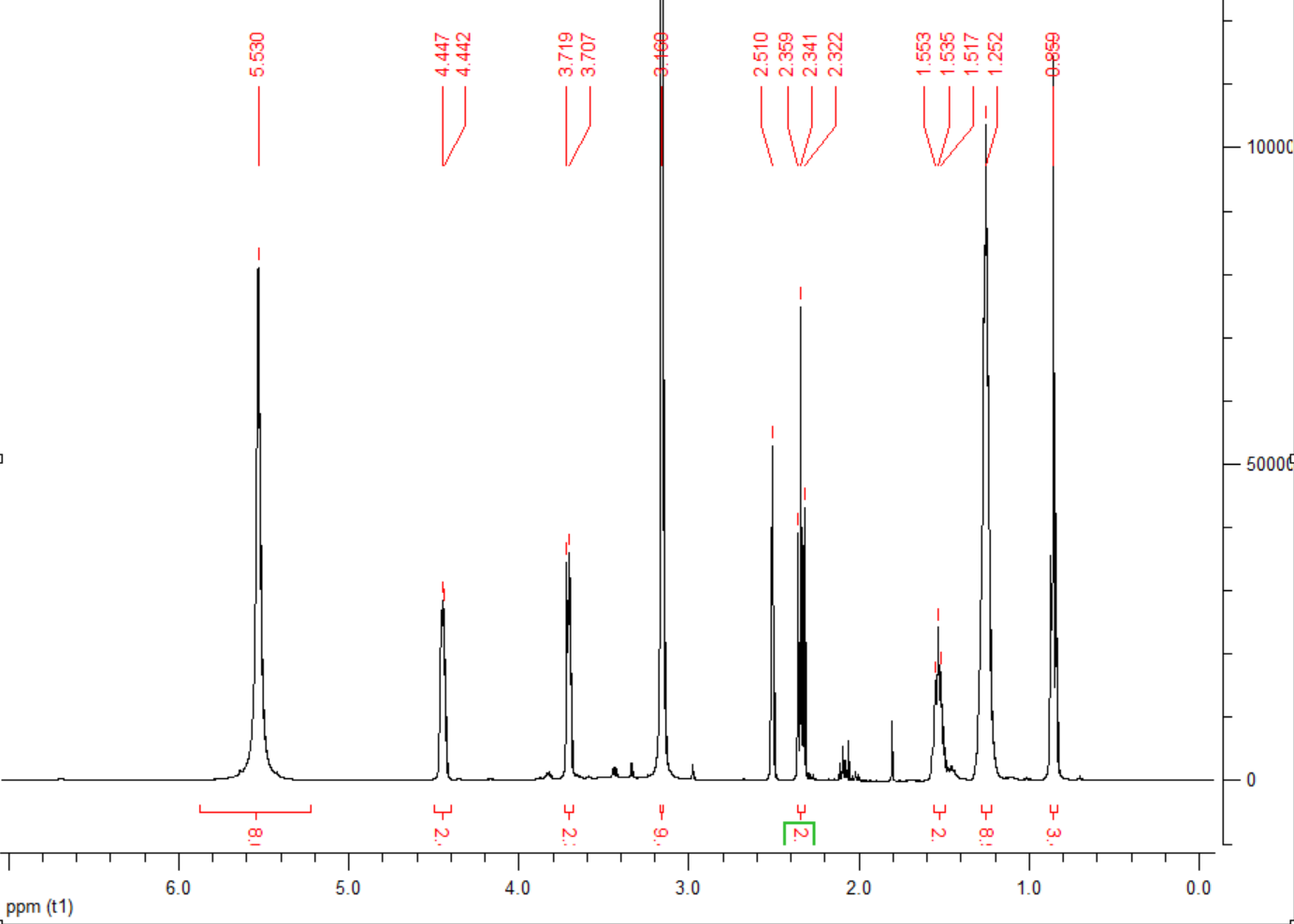


Fig. S10. ^1^H NMR of choline chloride octanoate-urea (1:2, v/v).


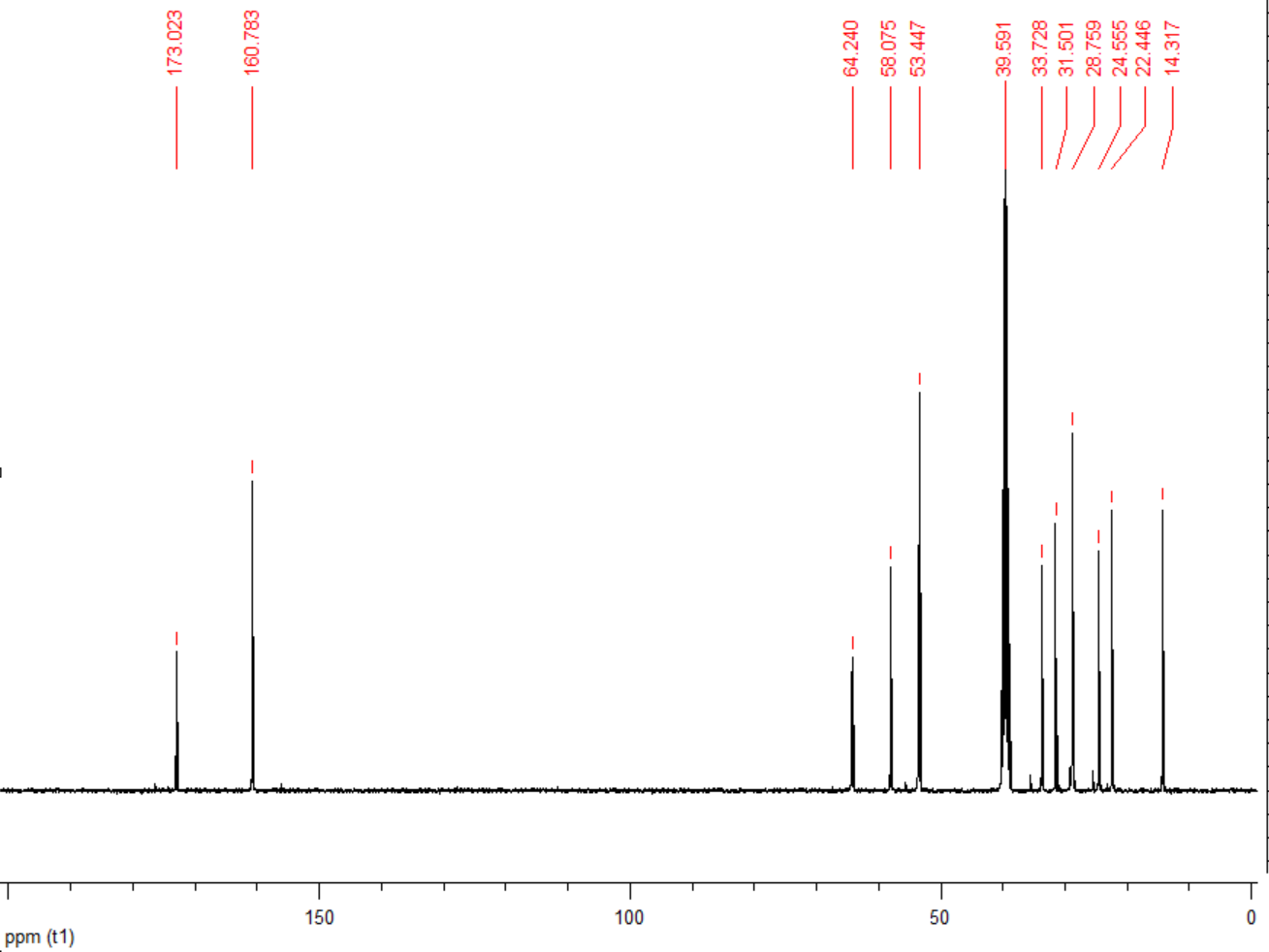


Fig. S11. ^13^C NMR of choline chloride octanoate-urea (1:2, v/v).


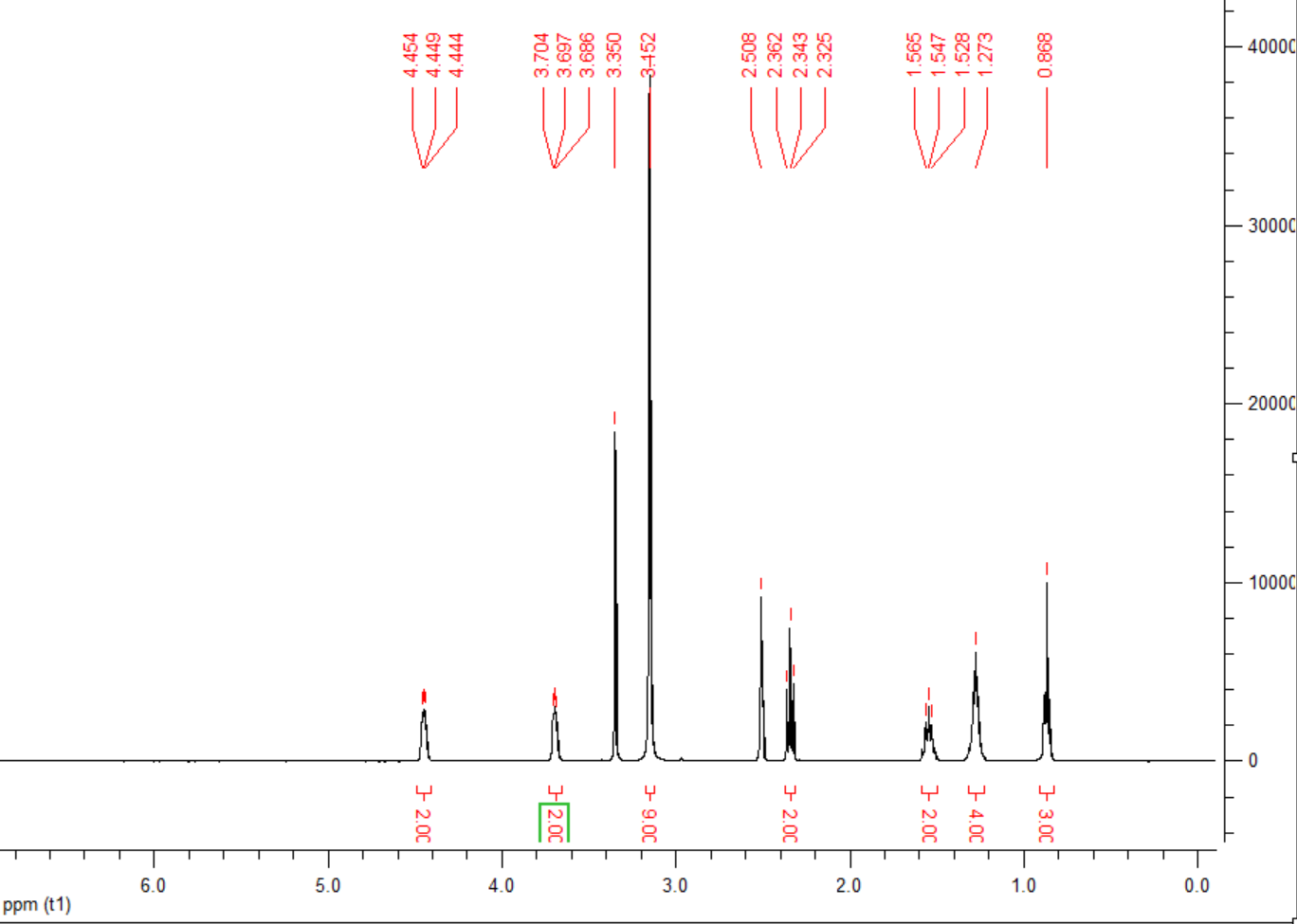


Fig. S12. ^1^H NMR of choline chloride hexanoate.


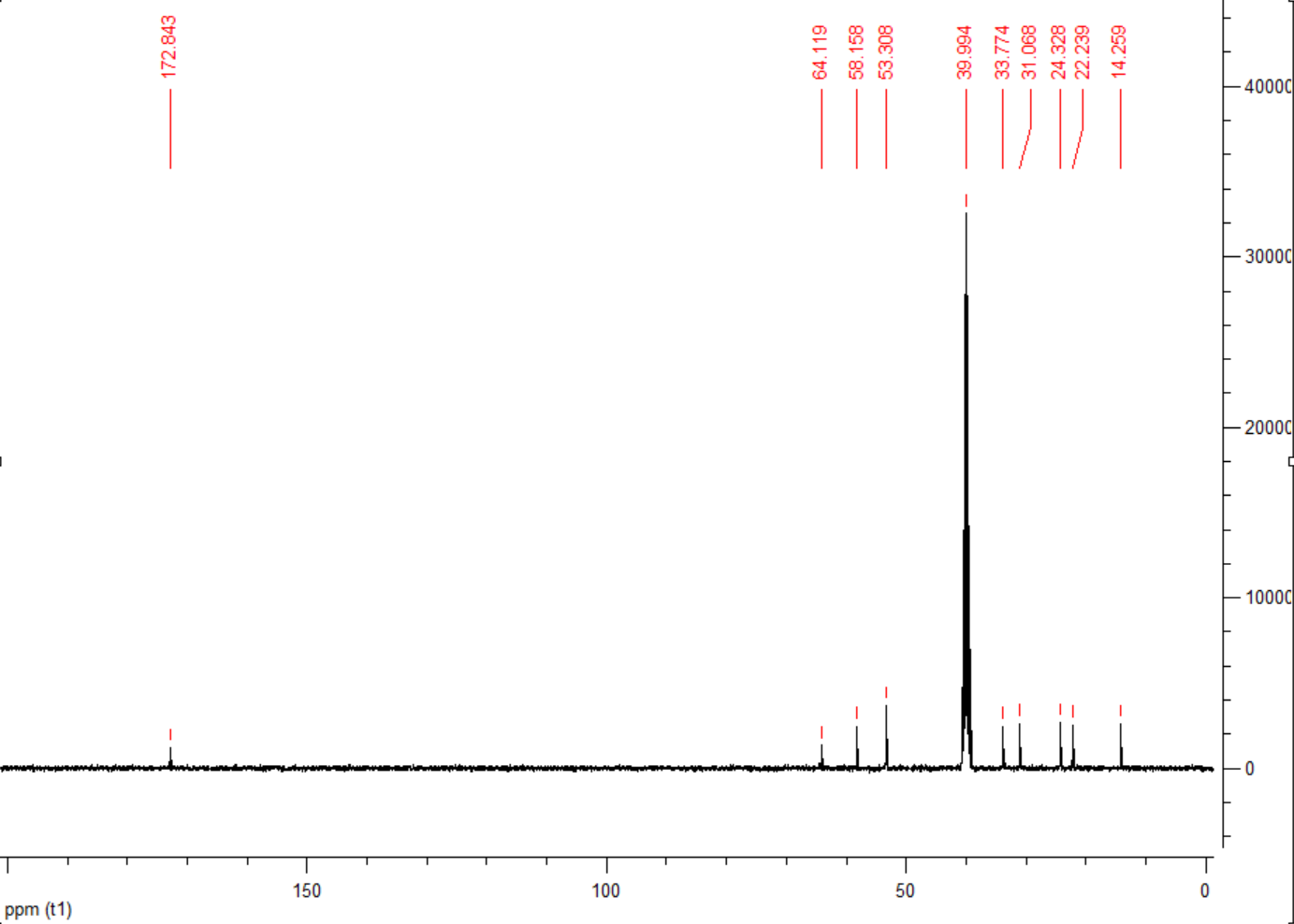


Fig. S13. ^13^C NMR of choline chloride hexanoate.

**
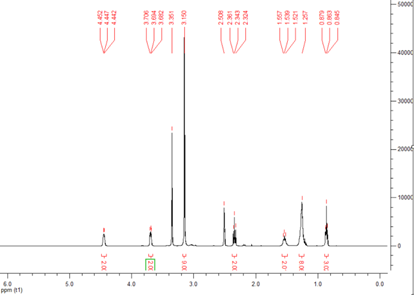
**

Fig. S14. ^1^H NMR of choline chloride octanoate.


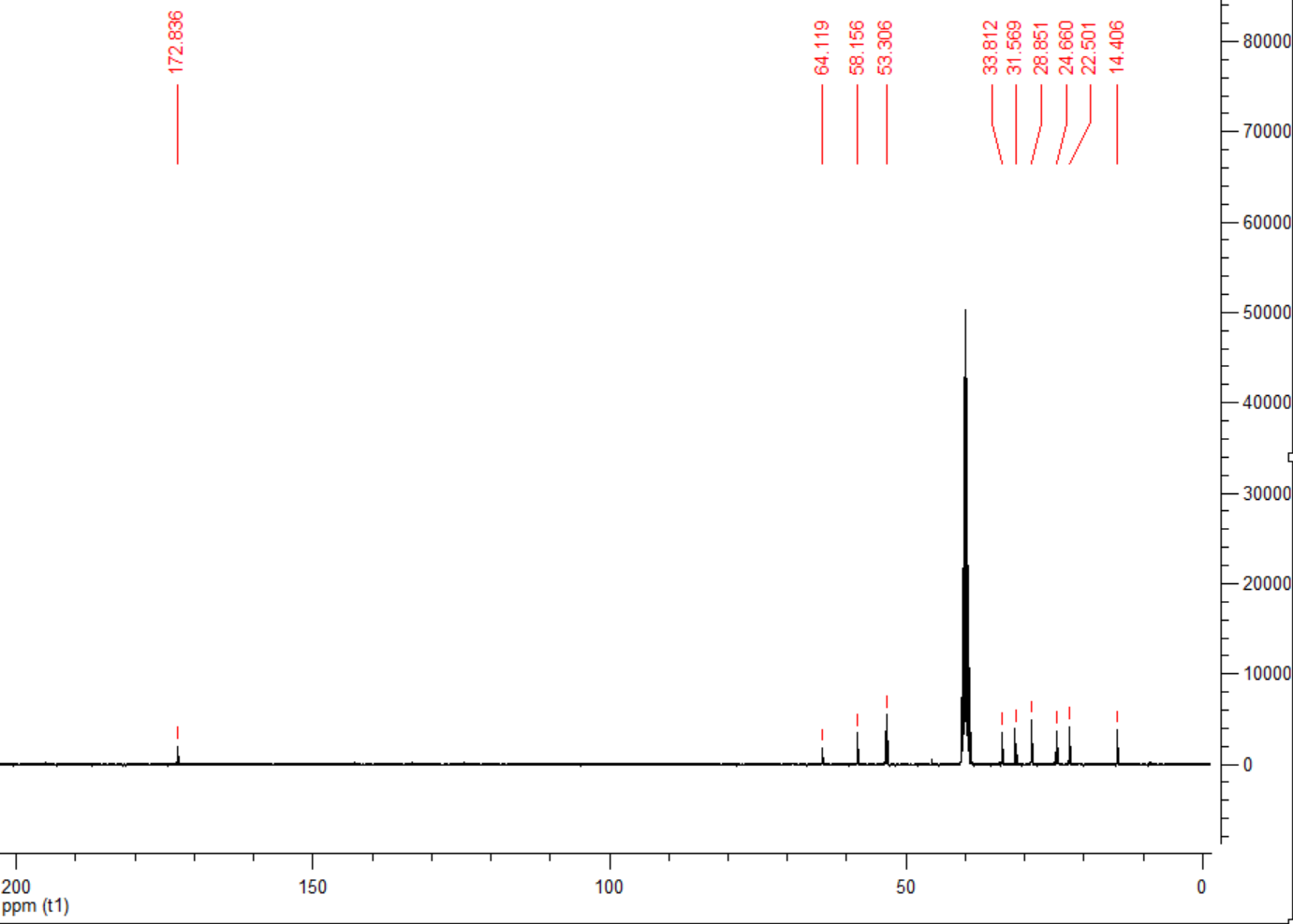


Fig. S15. ^13^C NMR of choline chloride octanoate.

**HRMS spectra**


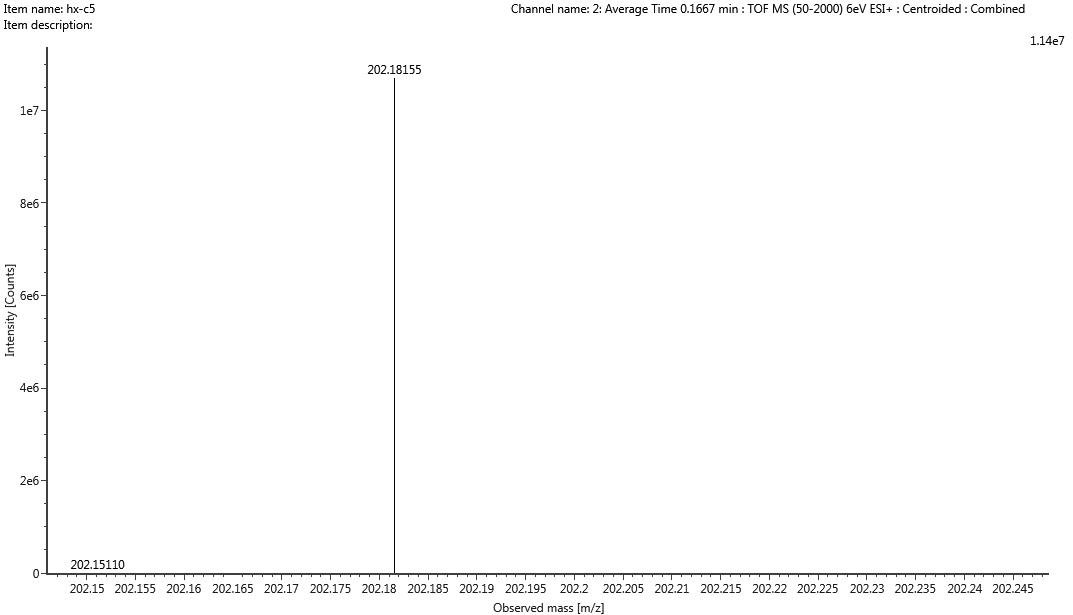


Fig. S16. HRMS of choline chloride hexanoate.


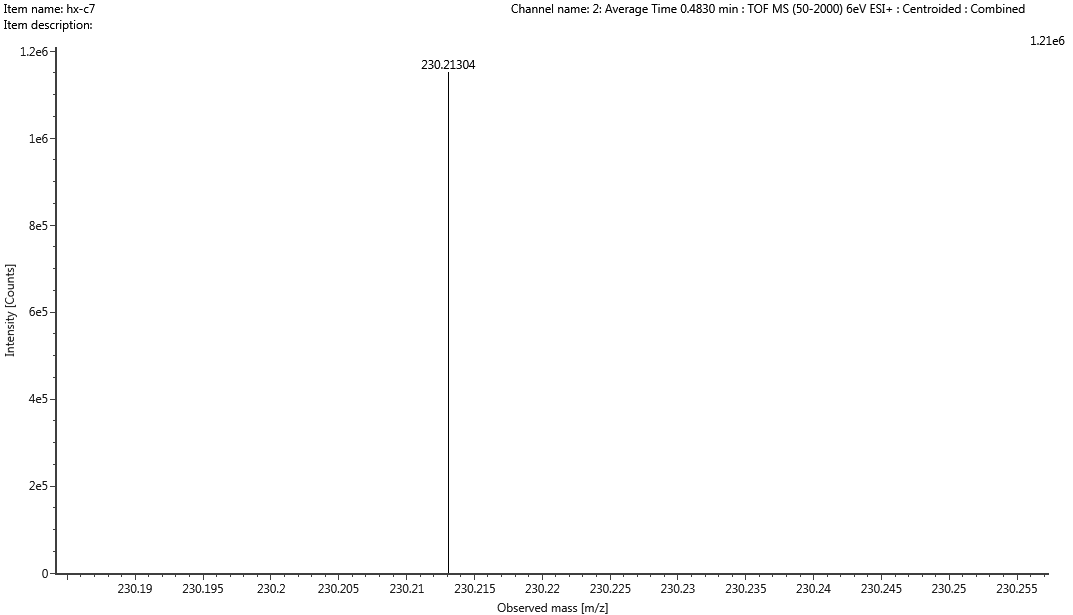


Fig. S17. HRMS of choline chloride octanoate.

**FT-IR spectra**

**
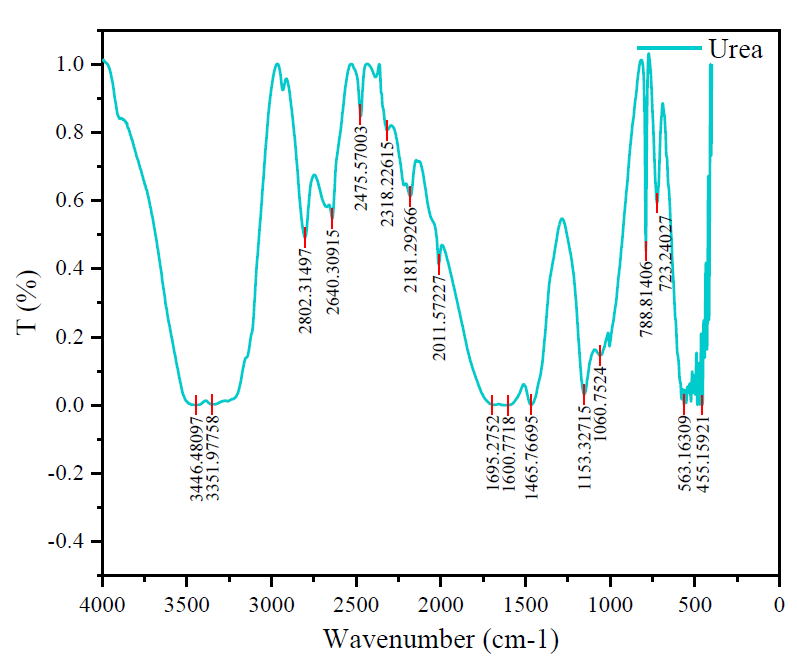
**

Fig. S18. FT-IR (TR) of urea

**
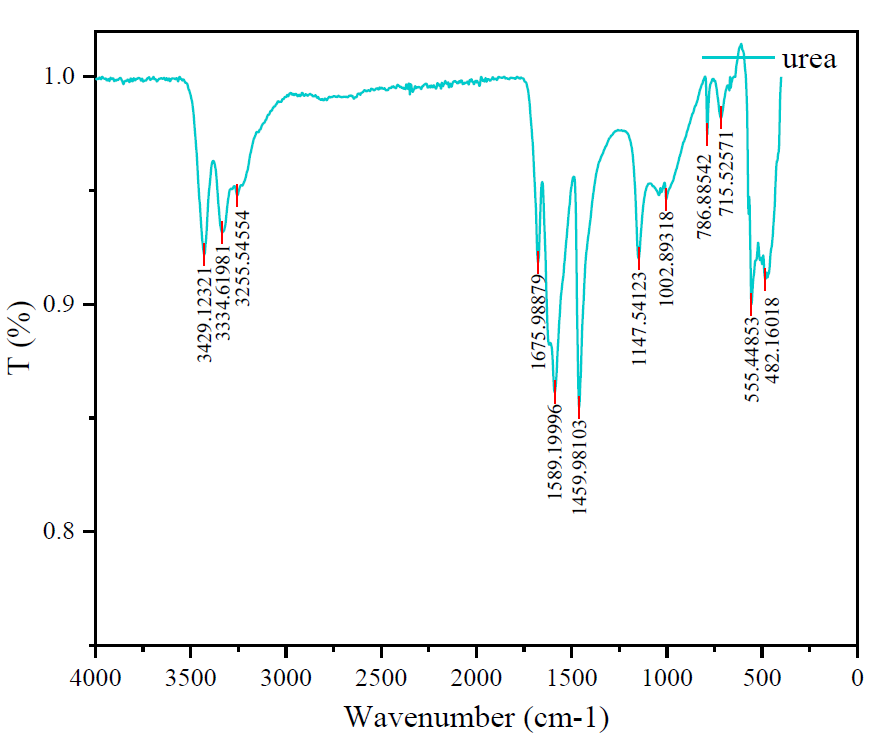
**

Fig. S19. FT-IR (ATR) urea

**
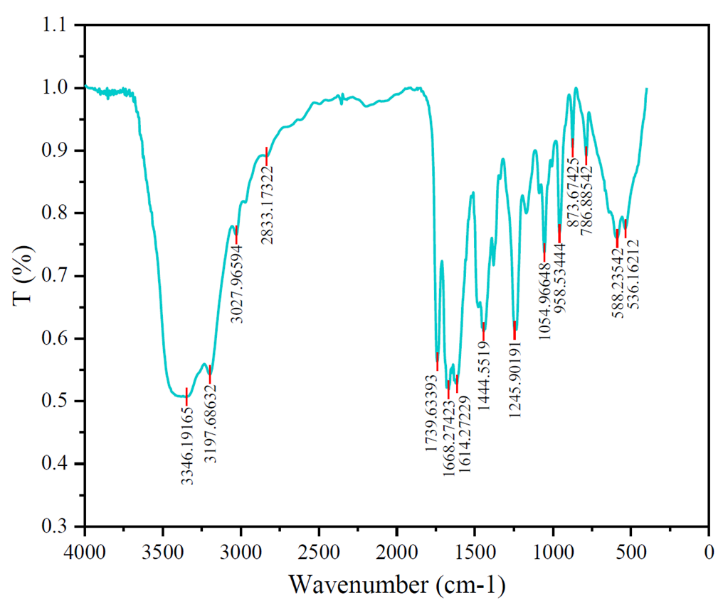
**

Fig. S20. FT-IR (TR) of acetylcholine chloride-urea (1:2, mol/mol).

**
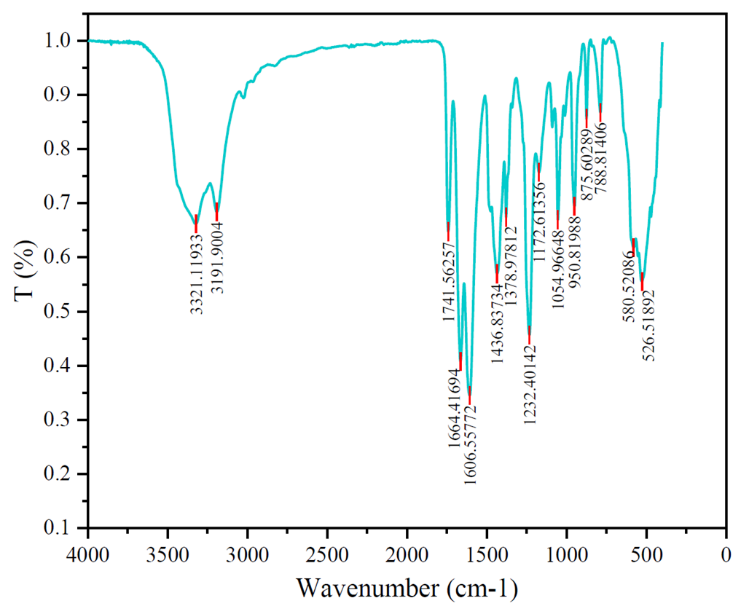
**

Fig. S21. FT-IR (ATR) of acetylcholine chloride-urea (1:2, mol/mol).

**
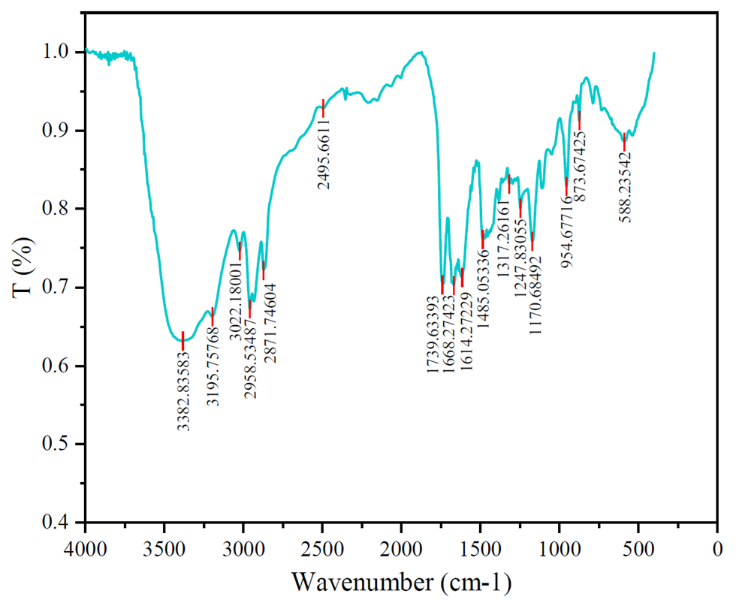
**

Fig. S22. FT-IR (TR) of choline chloride hexanoate-urea (1:2, mol/mol).

**
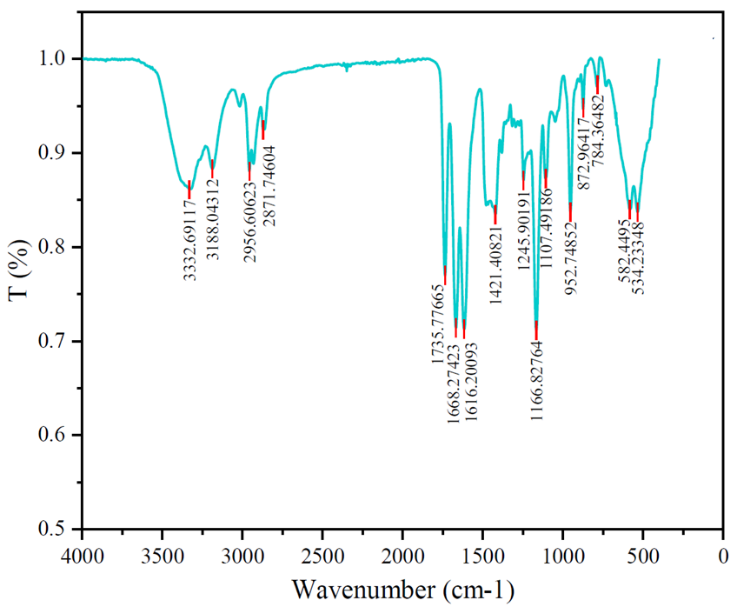
**

Fig. S23. FT-IR (ATR) of choline chloride hexanoate-urea (1:2, mol/mol).

**
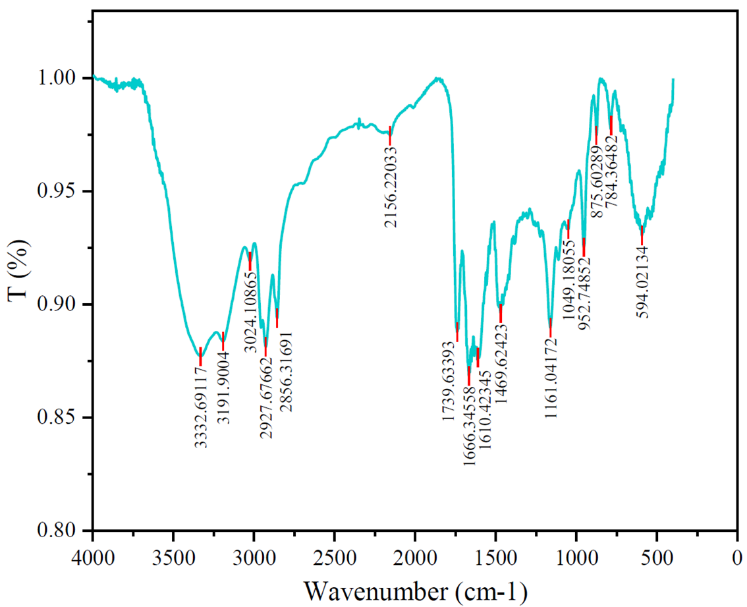
**

Fig. S24. FT-IR (TR) of choline chloride octanoate-urea (1:2, mol/mol).


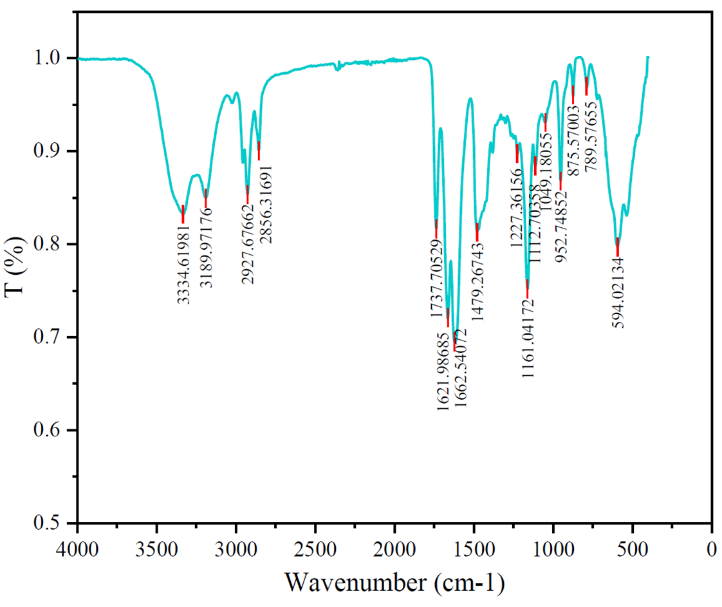


Fig. S25. FT-IR (ATR) of choline chloride octanoate-urea (1:2, mol/mol).

**
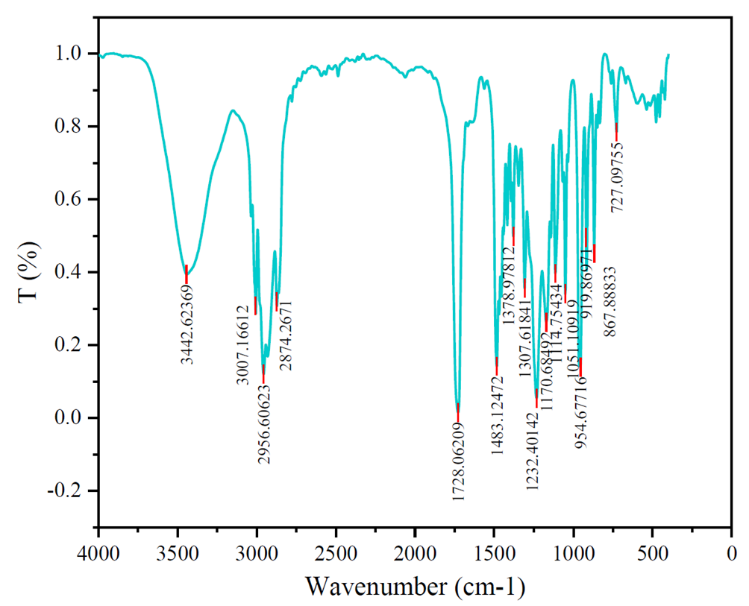
**

Fig. S26. FT-IR (TR) of choline chloride hexanoate.

**
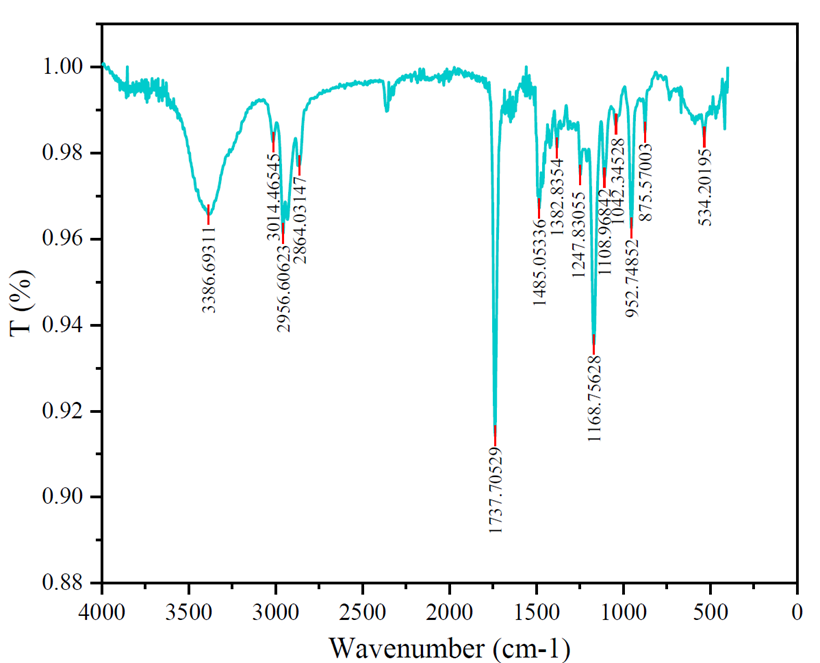
**

Fig. S27. FT-IR (ATR) of choline chloride hexanoate.

**
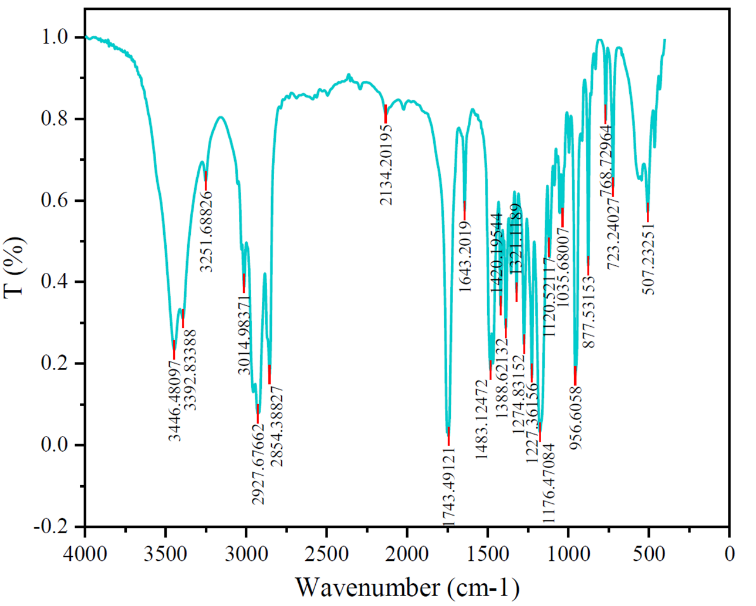
**

Fig. S28. FT-IR (TR) of choline chloride octanoate.

**
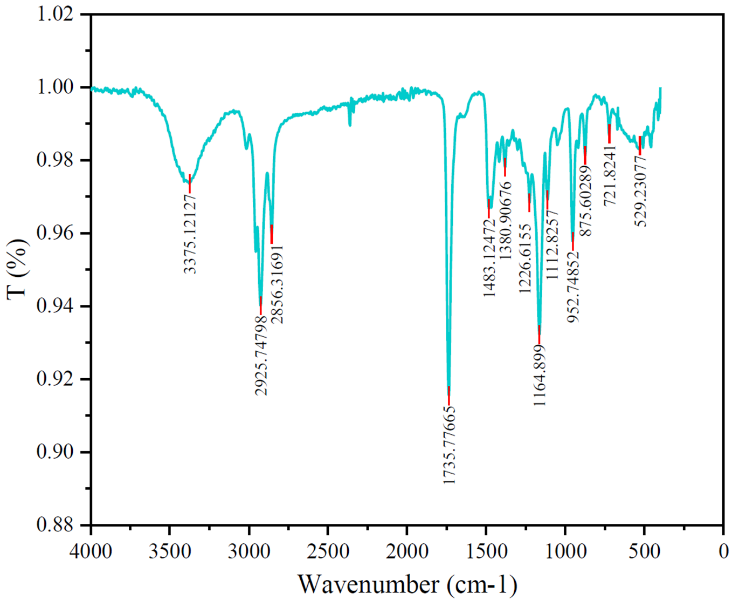
**

Fig. S29. FT-IR (ATR) of choline chloride octanoate.

**DSC curves**


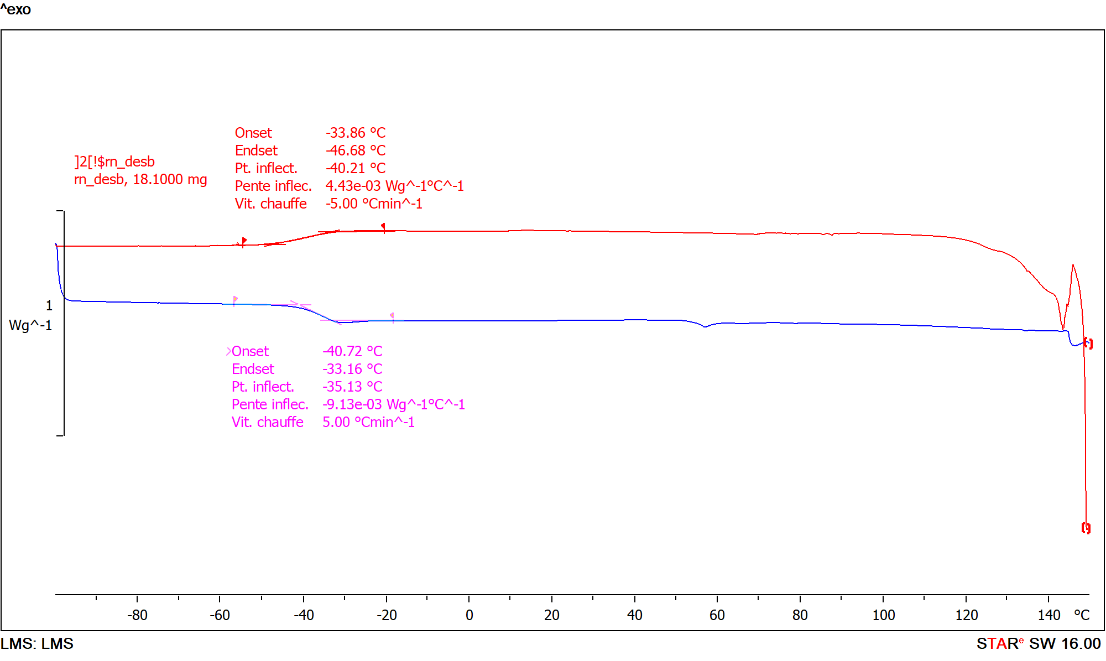


Fig. S30. DSC measurement of acetylcholine chloride-urea (1:2, mol/mol).


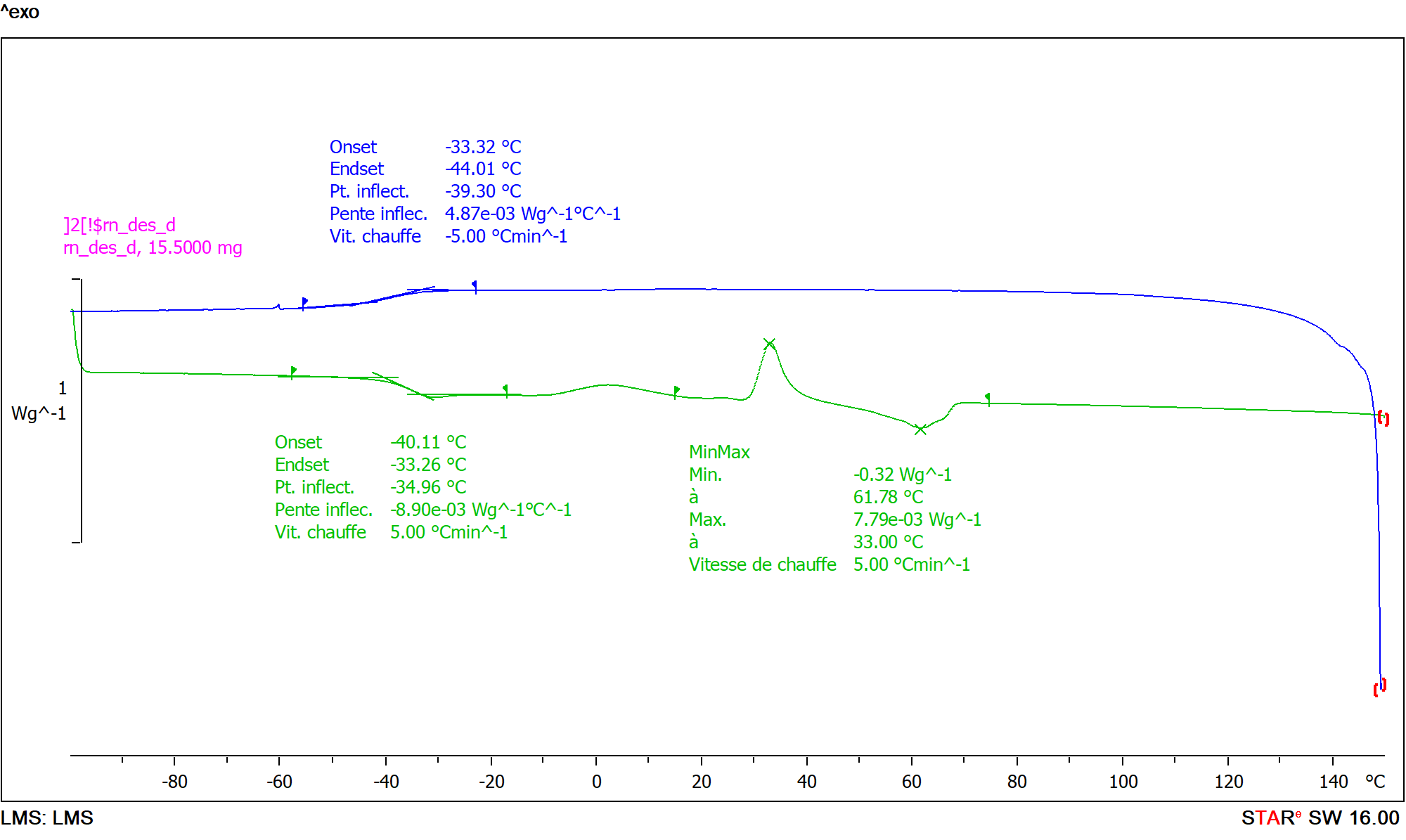


Fig. S31. DSC measurement of choline chloride hexanoate-urea (1:2, mol/mol).


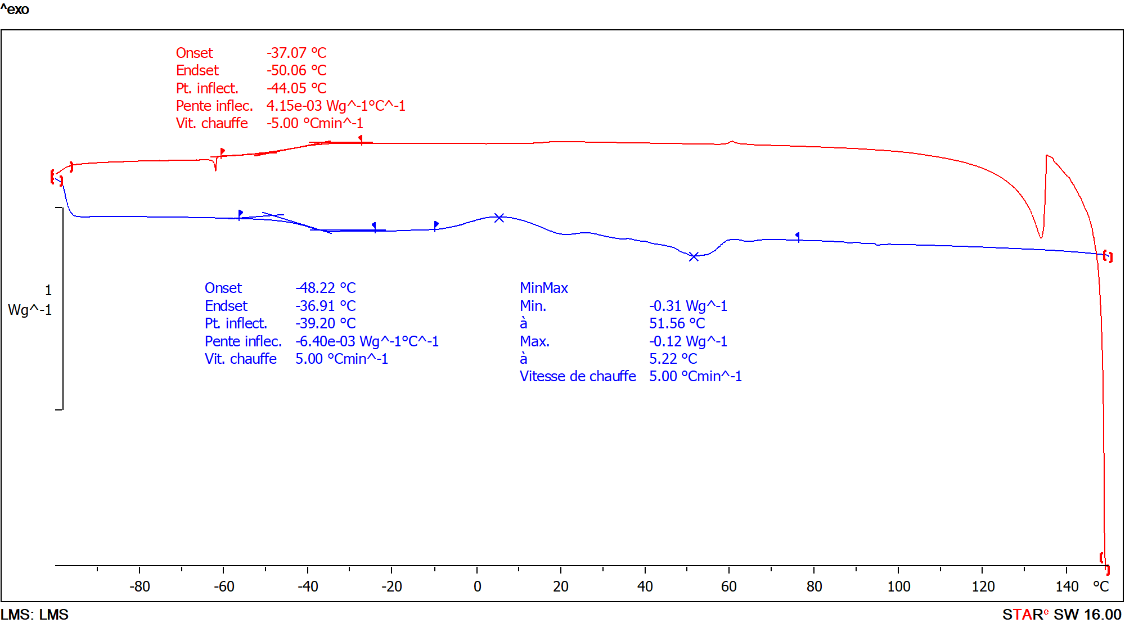


Fig. S32. DSC measurement of choline chloride octanoate-urea (1:2, mol/mol).

**SEM images**

Fig. S33. SEM images in secondary electron mode of the surface of the beads with corresponding EDS spectra, after corrosion; a) beads in 0.5M HCl corrosion media with choline chloride octanoate-urea (1:2, mol/mol) inhibitor, b) without inhibitor.

Compared to the initial surface state of the beads, both samples show significant degradation after exposure to the HCl solution. This degradation is characterized by the formation of cavities on the steel surface and the appearance of Cr-rich particles, ranging from 1 to 5 microns in diameter, as corrosion products. Notably, the density of these corrosion products is considerably higher in sample b) than in sample a).

**Reference**

1. P. Abbott, G. Capper, D. L. Davies, R. K. Rasheed, V. Tambyrajah, Novel solvent properties of choline chloride/urea mixtures, *Chem. Commun.* **2003**, 70-71. DOI: 10.1039/b210714g
